# Supplementary material for: Safety of inadvertent administration of live zoster vaccine to immunosuppressed individuals in a UK-based observational cohort analysis
Source: BMJ Open. 2020 Jan 29;10(1):e034886. doi: 10.1136/bmjopen-2019-034886 (PMC7045073; doi:10.1136/bmjopen-2019-034886)
Supplement: Supplementary data [file bmjopen-2019-034886supp001.pdf]

Appendices

Appendix A1: Approach to defining age-eligible cohorts for zoster vaccination

CPRD data are anonymised and includes year of birth but not day or month. This is a challenge for identifying the age-cohorts eligible for vaccination. Age eligibility for zoster vaccination has differed for each year of the vaccination programme since its introduction, as follows:

- *2013/14*: those aged 70 or 79 years on 1st Sept 2013;
- *2014/15:* those aged 70, 78 or 79 years on 1st Sept 2014;
- *2015/16*: those aged 70, 71, 72, 78 or 79 years on 1st Sept 2015;
- *2016/17*: those aged 70, 71, 72, 73, 78 or 79 years on 1st Sept 2016.

The distribution of zoster vaccine eligibility according to year of birth is detailed in Table A1, below. We included the following age cohorts over the four years of the study to ensure that at least 67% of the cohort was age-eligible in each year:

- Study year 1: 2013/14: years of birth 1943, 1934
- Study year 2: 2014/15: years of birth 1944, 1935, 1936
- Study year 3: 2015/16: years of birth 1943*, 1944*, 1945, 1936*, 1937
- Study year 4: 2016/17: years of birth 1943*, 1944*, 1945*, 1946, 1937*, 1938

  * Included if not previously vaccinated

Age-eligibility person years at risk adjustment

The rate of vaccination while immunosuppressed was calculated by adjusting the denominator person years at risk to account for age-eligibility uncertainty relating to unknown month of birth. For example, people born in 1943 were eligible if born prior to 2nd September (Table A1), so eligibility for those born in 1943 was assumed to be 67%. Therefore, the total person years at risk of people born in 1943 was adjusted by multiplying by 0.67. This process was followed for all years with partial age-eligibility.

For cumulative uptake analysis, those born in years with partial (67%) age-eligibility contributed 0.67 to the denominator.

Table A1: Summary of zoster vaccine eligibility by calendar year and year of birth

Year of birth	True day/month of birth	cohort	Year 1: 09/2013-08/2014 Age at 01/09	Year 1: Eligible?	Year 2: 09/2014-08/2015 Age at 01/09	Year 2: Eligible?	Year 3: 09/2015-08/2016 Age at 01/09	Year 3: Eligible?	Year 4: 09/2016-08/2017 Age at 01/09*	Year 4: Eligible?
1943	1st Jan-1st Sep	Routine	70	Eligible	71	Ineligible	72	Eligible	73	Eligible
1943	2nd Sep-31st Dec	Routine	69	Ineligible	70	Eligible	71	Eligible	72	Eligible
1944	1st Jan-1st Sep	Routine	69	Ineligible	70	Eligible	71	Eligible	72	Eligible
1944	2nd Sep-31st Dec	Routine	68	Ineligible	69	Ineligible	70	Eligible	71	Eligible
1945	1st Jan-1st Sep	Routine	68	Ineligible	69	Ineligible	70	Eligible	71	Eligible
1945	2nd Sep-31st Dec	Routine	67	Ineligible	68	Ineligible	69	Ineligible	70	Eligible
1946	1st Jan-1st Sep	Routine	67	Ineligible	68	Ineligible	69	Ineligible	70	Eligible
1946	2nd Sep-31st Dec	Routine	66	Ineligible	67	Ineligible	68	Ineligible	69	Ineligible*
1934	1st Jan-1st Sep	Catch up	79	Eligible	80	Ineligible	81	Ineligible	82	Ineligible
1934	2nd Sep-31st Dec	Catch up	78	Ineligible	79	Eligible	80	Ineligible	81	Ineligible
1935	1st Jan-1st Sep	Catch up	78	Ineligible	79	Eligible	80	Ineligible	81	Ineligible
1935	2nd Sep-31st Dec	Catch up	77	Ineligible	78	Eligible	79	Eligible	80	Ineligible
1936	1st Jan-1st Sep	Catch up	77	Ineligible	78	Eligible	79	Eligible	80	Ineligible
1936	2nd Sep-31st Dec	Catch up	76	Ineligible	77	Ineligible	78	Eligible	79	Eligible
1937	1st Jan-1st Sep	Catch up	76	Ineligible	77	Ineligible	78	Eligible	79	Eligible
1937	2nd Sep-31st Dec	Catch up	75	Ineligible	76	Ineligible	77	Ineligible	78	Eligible
1938	1st Jan-1st Sep	Catch up	75	Ineligible	76	Ineligible	77	Ineligible	78	Eligible
1938	2nd Sep-31st Dec	Catch up	74	Ineligible	75	Ineligible	76	Ineligible	77	Ineligible*

* From April 2017, rules changed so patients became eligible on the day they turned 70yrs (routine) or 78 years (catch up); those with existing eligibility who missed vaccination could still be offered the vaccine.

Appendix A2: Summary of definitions of periods of immunosuppression

Immunosuppression category	Category includes	Code types used to identify immunosuppression	Time period defined as immunosuppressed following each record
Haematological malignancies	Leukaemias, lymphomas, other lymphoproliferative disorders	Read v2 codes<br>ICD-10 codes[1]	24 months
HIV/AIDS		Read v2 codes<br>ICD-10 codes[1]	Permanent
Cellular immune deficiencies	Permanent	Read v2 codes<br>ICD-10 codes[1]	Permanent
	Aplastic anaemia	Read v2 codes<br>ICD-10 codes[1]	24 months
	Other/unspecified cellular immune deficiences	Read v2 codes<br>ICD-10 codes[1]	90 days
Bone marrow transplants	Allogenic or autologous stem cell transplant	Read v2 codes<br>ICD-10 codes[1]<br>OPCS procedure codes[1]	24 months
Immunosuppressive therapies for solid organ transplant	Solid organ transplant	Read v2 codes<br>ICD-10 codes[1]<br>OPCS procedure codes[1]	Permanent
Chemotherapy or radiotherapy		Read v2 codes<br>Prescriptions<br>ICD-10 codes[1]<br>OPCS procedure codes[1]	1 year unless a record of end of therapy, in which case 6 months
Oral corticosteroids	Short term high-dose corticosteroids >40mg prednisolone per day for more than 1 week	Prescriptions	3 months
	Long term lower dose corticosteroids (>20mg prednisolone per day for more than 14 days	Prescriptions	3 months
Biological therapies	Abatacept, adalimumab, aflibercept, alemtuzumab, anakinra, apremilast, thymoglobulin, baricitinib, basiliximab, belatacept, bevacizumab, bortezomib, brentuximab, canakinumab, cetuximab, certolizumab, daclizumab, dasatinib, eculizumab, etanercept, everolimus, fingolimod, golimumab, idelalisib, imatinib, infliximab, ipilimumab, natalizumam, nilotinib, nivolumab, obinutuzumab, ofatumumab, panitumumab, pembrolizumab, pertuzumab, rituximab, secukinumab, temsirolimus, tocilizumab, tofacitinib, trastuzumab, ustekinumab, vedolizumab.	Prescriptions	3 months before first ever prescription[2] and 12 months following each prescription

Other immunosuppressant medications	Methotrexate >25mg per week; Azathioprine >3.0mg/kg/day; 6-mercaptopurine >1.5mg/kg/day; Corticosteroid injections; other DMARDS; other immunosuppressant medications	Prescriptions	3 months before first ever prescription[2] and 3 months following each prescription

1. For patients with available linkage to Hospital Episode Statistics (HES)
2. To reflect standard practice of initiation in secondary care.
