# Supplementary material for: Safety of inadvertent administration of live zoster vaccine to immunosuppressed individuals in a UK-based observational cohort analysis
Source: BMJ Open. 2020 Jan 29;10(1):e034886. doi: 10.1136/bmjopen-2019-034886 (PMC7045073; doi:10.1136/bmjopen-2019-034886)
Supplement: Author's manuscript [file bmjopen-2019-034886.draft_revisions.pdf]

BMJ Open is committed to open peer review. As part of this commitment we make the peer review history of every article we publish publicly available.

When an article is published we post the peer reviewers' comments and the authors' responses online. We also post the versions of the paper that were used during peer review. These are the versions that the peer review comments apply to.

The versions of the paper that follow are the versions that were submitted during the peer review process. They are not the versions of record or the final published versions. They should not be cited or distributed as the published version of this manuscript.

BMJ Open is an open access journal and the full, final, typeset and author-corrected version of record of the manuscript is available on our site with no access controls, subscription charges or pay-per-view fees (http://bmjopen.bmj.com).

If you have any questions on BMJ Open's open peer review process please email info.bmjopen@bmj.com

BMJ Open

Inadvertent administration of live zoster vaccine to immunosuppressed individuals: vaccine-related disease is a rare occurrence. An observational cohort analysis of electronic health records.

Journal:	*BMJ Open*
Manuscript ID	bmjopen-2019-034886
Article Type:	Original research
Date Submitted by the Author:	09-Oct-2019

Complete List of Authors:	Grint, Daniel; London School of Hygiene and Tropical Medicine, Infectious Disease Epidemiology<br>McDonald, Helen; London School of Hygiene and Tropical Medicine, Infectious Disease Epidemiology<br>Walker, Jemma; London School of Hygiene and Tropical Medicine, Faculty of Epidemiology and Population Health; Public Health England, Statistics, Modelling and Economics Department<br>Amirthalingam, Gayatri; Public Health England, Immunisation and Countermeasures Division<br>Andrews, Nick; Public Health England, Statistics, Modelling and Economics Department; London School of Hygiene and Tropical Medicine, Infectious Disease Epidemiology<br>Thomas, Sara; London School of Hygiene and Tropical Medicine, Infectious Disease Epidemiology

Keywords:	Infectious diseases & infestations < DERMATOLOGY, Geriatric dermatology < DERMATOLOGY, IMMUNOLOGY, Public health < INFECTIOUS DISEASES, PUBLIC HEALTH, Adverse events < THERAPEUTICS

SCHOLARONE™
Manuscripts

I, the Submitting Author has the right to grant and does grant on behalf of all authors of the Work (as defined in the below author licence), an exclusive licence and/or a non-exclusive licence for contributions from authors who are: i) UK Crown employees; ii) where BMJ has agreed a CC-BY licence shall apply, and/or iii) in accordance with the terms applicable for US Federal Government officers or employees acting as part of their official duties; on a worldwide, perpetual, irrevocable, royalty-free basis to BMJ Publishing Group Ltd ("BMJ") its licensees and where the relevant Journal is co-owned by BMJ to the co-owners of the Journal, to publish the Work in this journal and any other BMJ products and to exploit all rights, as set out in our licence.

The Submitting Author accepts and understands that any supply made under these terms is made by BMJ to the Submitting Author unless you are acting as an employee on behalf of your employer or a postgraduate student of an affiliated institution which is paying any applicable article publishing charge ("APC") for Open Access articles. Where the Submitting Author wishes to make the Work available on an Open Access basis (and intends to pay the relevant APC), the terms of reuse of such Open Access shall be governed by a Creative Commons licence – details of these licences and which Creative Commons licence will apply to this Work are set out in our licence referred to above.

Other than as permitted in any relevant BMJ Author's Self Archiving Policies, I confirm this Work has not been accepted for publication elsewhere, is not being considered for publication elsewhere and does not duplicate material already published. I confirm all authors consent to publication of this Work and authorise the granting of this licence.

For peer review only

Inadvertent administration of live zoster vaccine to immunosuppressed individuals: vaccine-related disease is a rare occurrence

An observational cohort analysis of electronic health records

Authors: Daniel J Grint (0000-0001-8001-6544),[1] Helen I McDonald (0000-0003-0576-2015),[1] Jemma L Walker,[1,2] Gayatri Amirthalingam,[3] Nick Andrews,[1,2] Sara L Thomas.[1]

1. Department of Infectious Disease Epidemiology, London School of Hygiene & Tropical Medicine, London, UK

Daniel J Grint assistant professor, Helen I McDonald honorary research fellow, Jemma L Walker research fellow, Nick Andrews honorary professor, Sara L Thomas professor (retired).

2. Statistics, Modelling and Economics Department, Public Health England, London, UK

Jemma L Walker statistician, Nick Andrews senior statistician.

3. Immunisation and Countermeasures Division, National Infection Service, Public Health England, London, UK

Gayatri Amirthalingam consultant epidemiologist.

Correspondence to: Helen McDonald, London School of Hygiene & Tropical Medicine, Keppel Street, London, WC1E 7HT. Helen.mcdonald@lshtm.ac.uk Tel: 020 7612 7921 Fax: N/A

Article type: Research

For peer review only - http://bmjopen.bmj.com/site/about/guidelines.xhtml

For peer review only

For peer review only - http://bmjopen.bmj.com/site/about/guidelines.xhtml

Abstract

Objectives:

To investigate the safety of live attenuated varicella zoster vaccination when administered to immunosuppressed individuals.

Design:

Prospective observational cohort study.

Setting:

The study used anonymised data from the Clinical Practice Research Datalink (CPRD), comprising a representative sample of routinely collected primary care data in England between 2013 and 2017, and linked Hospital Episode Statistics (HES) data.

Participants:

168,767 individuals age-eligible for varicella zoster vaccination registered at a general practice in England contributing data to CPRD.

Main outcome measures:

Electronic health records of conditions indicating immunosuppression, administration of zoster vaccination, diagnoses of specific zoster-related disease and non-specific rash compatible with zoster disease.

Results:

Between 1st September 2013 and 31st August 2017, a period of immunosuppression was identified for 9,093/168,767 (5·4%; 95% CI: 5·3-5·5%) individuals age-eligible for zoster vaccination. The overall rate of vaccination while immunosuppressed was 1,742/5,251 (33·2 per 100 adjusted person years at risk; 95% CI: 31·9-34·5). Follow-up of the 1,742 individuals who were inadvertently vaccinated while immunosuppressed identified only two cases of shingles in primary care data within 8 weeks of vaccination (0·1%; 95% CI: 0·01-0·4%), neither with a related hospital admission.

Conclusions:

Despite evidence of inadvertent vaccination of immunosuppressed individuals with live zoster vaccination, there is a lack of evidence of severe consequences including hospitalisation. This should reassure primary care staff and encourage vaccination of mildly immunosuppressed individuals who

For peer review only - http://bmjopen.bmj.com/site/about/guidelines.xhtml

do not meet current thresholds for contraindication. These findings support a review of the extent to which live zoster vaccination is contraindicated among the immunosuppressed.

For peer review only

For peer review only - http://bmjopen.bmj.com/site/about/guidelines.xhtml

Summary box

What is already known on this topic:

- Immunosuppression is associated with a high burden of shingles ('zoster') and its complications.
- High-profile case reports of fatal vaccine-related disease among severely immunosuppressed individuals cause concern and may contribute to declining vaccine coverage.
- Previous studies among patients with selected immunosuppressive conditions have found low incidence of vaccine-related disease.

What this study adds:

- This study covered the full profile of causes of immunosuppression listed as contraindications to vaccination in UK national guidance.
- The study found no evidence of severe vaccine-related disease among 1,742 individuals who were inadvertently vaccinated with live zoster vaccine while immunosuppressed.

For peer review only

Introduction

Herpes zoster (shingles) is a common and painful disease caused by reactivation of varicella zoster virus (VZV), with debilitating complications including post-herpetic neuralgia. Live-attenuated zoster vaccine was introduced for immunocompetent adults aged 70-79 years in England in 2013, delivered in primary care.(1)

Immunosuppression is associated with a high burden of zoster and its complications,(2, 3) and there have been calls to consider vaccination for this population.(4) However, live zoster vaccine is currently contraindicated in immunosuppression.(1) High-profile case reports of fatal vaccine-related disease among severely immunosuppressed individuals have caused concern and may have contributed to declining vaccine coverage.(5, 6) Understanding the safety of live vaccination during immunosuppression is important to support guidance on use of the vaccine, to ensure that individuals who can safely benefit from the vaccine are enabled to do so. A new recombinant vaccine is becoming available but stock is limited. Understanding the safety of live vaccination for typical causes of immunosuppression will be important to prioritise use of limited supplies of the new recombinant vaccine, and to understand any safety risk from potential product confusion if in future there may be parallel use of live and recombinant vaccines to different patient groups.

This study aimed to investigate the frequency and consequences of live zoster vaccination during immunosuppression among a large UK cohort from 2013 to 2017.

For peer review only

For peer review only - http://bmjopen.bmj.com/site/about/guidelines.xhtml

Methods

Data source

This study used anonymised data from the Clinical Practice Research Datalink (CPRD). The data include information on year of birth, medical diagnoses (version 2 Read codes), prescriptions and vaccinations. For 60% of individuals, records are pre-linked to anonymised hospitalisation data (Hospital Episode Statistics, HES). HES-linked data for inpatient admissions (International Classification of Disease, ICD-10 codes) and procedures (OPCS-4 Classification of Procedures codes) were used to supplement identification of immunosuppressed individuals and zoster disease.

Study population

Immunosuppressed individuals age-eligible for zoster vaccination, active in CPRD from September 2013 to August 2017 and registered with a CPRD practice for at least a year before study entry, were included.

Age eligibility for zoster vaccination has differed each year since the vaccination introduction. As month of birth was not available, individuals born in years for which ≥67% of the population would have been eligible for vaccination were included (Appendix A1).

Periods of immunosuppression were identified using Read codes and prescription records from CPRD, plus ICD-10 codes and OPCS codes in linked HES data. Immunosuppression was defined based on contraindications to live zoster vaccination described in national guidance.(1) The time periods assigned to each immunosuppressing condition or medication type, and dose thresholds for relevant medications, are described in Appendix A2. For prescription records missing dose, the median was imputed according to category of age and sex, in line with previous zoster studies.(3)

Vaccination status and vaccine-related varicella zoster disease

For peer review only - http://bmjopen.bmj.com/site/about/guidelines.xhtml

Individuals were followed to the first positive record of zoster vaccination. If this indicated that the vaccine was delivered by another health care provider the individual was excluded from the cohort, as timing of vaccination could not be determined (N=29).

Evidence of varicella zoster disease was assessed during the 8 weeks following a vaccination given while immunosuppressed. For the primary analysis, only specific diagnoses of VZV disease were included. Sensitivity analysis also included any rash that was unspecified or compatible with VZV, and acute encephalitis of unspecified aetiology. For individuals with HES-linkage, any diagnosis recorded within 8 weeks following vaccination was used to supplement identification of varicella-zoster disease. Codelists are available at https://datacompass.lshtm.ac.uk/1336/.

Statistical methods

An open prospective cohort study design was used whereby individuals exited and re-entered the cohort according to time-varying immunosuppression status. Follow-up started at 1st September of the study year in which the individual was age-eligible for vaccination and ended at the earliest of death, date of transfer out of practice, practice last collection date, elapsed age-eligibility for zoster vaccination, resolution of immunosuppression, or 31st August 2017.

The vaccination rate was calculated by total person years at risk (PYAR) while immunosuppressed with adjustment to account for age-eligibility uncertainty from unknown month of birth (Appendix A1). Cumulative uptake of zoster vaccine was computed stratified by treatment cohort and programme year.

The number of vaccinated immunosuppressed individuals who developed VZV disease in the subsequent eight weeks was described. In sensitivity analysis, disease in the first week following vaccination was excluded.

Statistical analysis was performed using STATA version 14·2 and SAS version 9.4.

Ethics approval

For peer review only - http://bmjopen.bmj.com/site/about/guidelines.xhtml

The study was approved by the Independent Scientific Advisory Group of the CPRD (ISAC reference 18_218) and the London School of Hygiene and Tropical Medicine Ethics Committee (reference 16298).

Patient and public involvement

Patients were not involved in this study.

For peer review only

For peer review only - http://bmjopen.bmj.com/site/about/guidelines.xhtml

Results

Between 1st September 2013 and 31st August 2017 data were available for 168,767 individuals age-eligible for vaccination, of whom 89,416 (53·0%) were female and 76,337 (45·2%) in the catch-up cohort. A period of immunosuppression while age-eligible for vaccination was identified for 9,093/168,767 (5·4%; 95% CI: 5·3-5·5%).

1,742 individuals were vaccinated during a period of immunosuppression. Adjusting person years at risk while immunosuppressed to account for age-eligibility uncertainty, the overall rate of vaccination during immunosuppression was 1,742/5,251 (33·2 per 100 adjusted person years at risk; 95% CI: 31·9-34·5). Figure 1 shows the cumulative uptake of zoster vaccine by programme year overall, in maiden years of eligibility and by cohort. Cumulative uptake was higher in programme years 3 and 4 when restricted to maiden years of eligibility. Cumulative uptake was highest in programme years 1 and 2 for both the routine and catch-up cohorts.

Among those vaccinated while immunosuppressed the most common underlying cause was chemotherapy (55·3%), followed by non-steroid drug use (13·2%), multiple indications (11·4%), and steroid drug use (11·3%). 47 (2·7%) had a permanent cause of immunosuppression. 368 (21·1%) were immunosuppressed for the duration of follow-up; median follow-up 32·2 months (IQR: 19·7-48·0). Vaccination took place during the final four weeks of a defined period of immunosuppression for 138/1,742 (7·9%).

In the eight weeks following vaccination 2/1,742 (0·1%; 95% CI: 0·01-0·4%) had a diagnosis of shingles recorded in primary care. Both individuals had HES-linkage available, however, neither had a related hospital admission. One of these cases occurred within 7 days of vaccination.

Using a broader definition including non-specific rash or encephalitis identified a further 23 possible cases of VZV disease (in total 25/1,742 (1·4%; 95% CI: 0·9-2·1%)). All additional possible cases were

For peer review only - http://bmjopen.bmj.com/site/about/guidelines.xhtml

For peer review only

instances of unspecified rash in primary care. In total, 22/25 (88%) had HES-linkage available, however, none had a related hospital admission recorded. Five of the broader definition cases occurred within 7 days of vaccine administration.

Chemotherapy was the cause of immunosuppression for the majority of cases who developed specific or non-specific vaccine-related disease (15/25 (60%)). The remaining cases included immunosuppression by oral steroid use, other immunosuppressant medications, leukaemia, and organ transplant.

For peer review only

For peer review only - http://bmjopen.bmj.com/site/about/guidelines.xhtml

Discussion

This is the first study to investigate the safety of live zoster vaccination across the range of contraindicating immunosuppressive conditions. Our study identified 1,742 individuals vaccinated while immunosuppressed and two subsequent cases of shingles, with no related hospitalisations.

A key strength of this study is the thorough ascertainment of immunosuppression and VZV disease using linked primary and secondary care data for a large, representative cohort with a range of immunosuppressive conditions.

The study has limitations. A key limitation is that month of birth was not available for precise identification of age-eligibility. If immunosuppressed individuals in a birth cohort with 67% eligibility were vaccinated while not age-eligible, rates of vaccination in immunosuppression would be overestimated. There also remains uncertainty in defining time-periods of immunosuppression and in imputing missing dose data for medications, which may result in under- or over-ascertainment of immunosuppression.

It is possible that zoster disease may have been under-ascertained, despite the use of both primary and secondary care records and a broad case definition. Conversely, as this population has a high baseline risk of naturally occurring shingles, we may have over-estimated the risk attributable to vaccination, particularly when including cases within 7 days of vaccination.

Finally, while our definitions of immunosuppression followed national guidance, we could not replace clinical judgement on severity or timing of immunosuppression.(1) Clinicians may have selectively, rather than inadvertently, vaccinated individuals at lower risk of vaccine-related disease. However, the most frequent contraindication was chemotherapy and only a small proportion of vaccinations occurred towards the end of a period of immunosuppression.

Our findings are consistent with previous studies of live zoster vaccination among patients with selected immunosuppressive conditions.(7-9) Studies showing that VZV-specific immunity may persist

For peer review only - http://bmjopen.bmj.com/site/about/guidelines.xhtml

or even be boosted by vaccination during cell-mediated immunosuppression,(10-12) further support the plausibility of residual immunity against vaccine-related disease despite immunosuppression.

Rates of zoster vaccination during immunosuppression were high, and similar in routine and catch-up cohorts. Analysis restricted to maiden years of eligibility suggests that the apparent decline in vaccination rates after year 2 is partly a cohort effect, whereby people who were unvaccinated despite previous eligibility were less likely to be vaccinated subsequently. Increasingly detailed guidance over time may also have helped reduce inadvertent vaccination.

Among this large cohort with a range of immunosuppressing conditions we found no evidence of severe disease following live zoster vaccination while immunosuppressed. This should reassure clinicians, and encourage vaccination of mildly immunosuppressed individuals who do not meet current thresholds for contraindication, especially in the current context of declining uptake of the national programme. These findings support a review of the extent to which live zoster vaccination is contraindicated among the immunosuppressed population. Further research is needed to identify any patient groups for whom recombinant zoster vaccine should be prioritised once stocks become available in the UK.

For peer review only

For peer review only - http://bmjopen.bmj.com/site/about/guidelines.xhtml

Role of the funding source

The research was funded by the National Institute for Health Research (NIHR) Health Protection Research Unit (HPRU) in Immunisation at the London School of Hygiene and Tropical Medicine in partnership with Public Health England (PHE). The funders had no role in the study design, collection analysis or interpretation of data, writing of the report nor the decision to submit the article for publication. The views expressed are those of the authors and not necessarily those of the NHS, the NIHR, the Department of Health and Social Care, or PHE.

Contributor and guarantor information:

SLT had the idea for the study. SLT, JLW, GA and NA obtained the data. All authors contributed to the design of the study. DJG analysed the data. All authors contributed to the interpretation of results. DJG and HIM drafted the manuscript and all authors revised it critically. All authors approved the final version to be published and agree to be accountable for all aspects of the work. DJG is the guarantor. The corresponding author attests that all listed authors meet authorship criteria and that no others meeting the criteria have been omitted.

Transparency declaration: DJG affirms that the manuscript is an honest, accurate, and transparent account of the study being reported; that no important aspects of the study have been omitted; and that any discrepancies from the study as originally planned (and, if relevant, registered) have been explained.

Competing interests declaration: All authors have completed the ICMJE uniform disclosure form at www.icmje.org/coi_disclosure.pdf and declare: DJG, JLW, NA, and SLT had financial support from the National Institute for Health Research (NIHR) Health Protection Research Unit (HPRU) in Immunisation for the submitted work; the Public Health England Immunisation Department has provided vaccine manufacturers with post-marketing surveillance reports on pneumococcal and meningococcal infection which the companies are required to submit to the UK Licensing Authority in compliance with their Risk Management Strategy, and a cost recovery charge is made for these

For peer review only

For peer review only - http://bmjopen.bmj.com/site/about/guidelines.xhtml

reports; no other financial relationships with any organisations that might have an interest in the submitted work in the previous three years; no other relationships or activities that could appear to have influenced the submitted work.

Licence for publication

The Corresponding Author has the right to grant on behalf of all authors and does grant on behalf of all authors, a worldwide licence to the Publishers and its licensees in perpetuity, in all forms, formats and media (whether known now or created in the future), to i) publish, reproduce, distribute, display and store the Contribution, ii) translate the Contribution into other languages, create adaptations, reprints, include within collections and create summaries, extracts and/or, abstracts of the Contribution, iii) create any other derivative work(s) based on the Contribution, iv) to exploit all subsidiary rights in the Contribution, v) the inclusion of electronic links from the Contribution to third party material where-ever it may be located; and, vi) licence any third party to do any or all of the above.

Data sharing statement

These data were obtained from the Clinical Practice Research Datalink, provided by the UK Medicines and Healthcare products Regulatory Agency. The authors' licence for using these data does not allow sharing of raw data with third parties. Information about access to Clinical Practice Research Datalink data is available here: https://www.cprd.com/research-applications

Codelists for this study are available at https://datacompass.lshtm.ac.uk/1336/.

For peer review only

For peer review only - http://bmjopen.bmj.com/site/about/guidelines.xhtml

References

1. Department of Health PHE. Immunisation against Infectious Disease (the Green Book). Chapter 28a: Shingles (herpes zoster) 2016. Available from: https://assets.publishing.service.gov.uk/government/uploads/system/uploads/attachment_data/file/503773/2905109_Green_Book_Chapter_28a_v3_0W.PDF

2. Yanni EA, Ferreira G, Guennec M, El Hahi Y, El Ghachi A, Haguinet F, et al. Burden of herpes zoster in 16 selected immunocompromised populations in England: a cohort study in the Clinical Practice Research Datalink 2000-2012. BMJ open. 2018;8(6):e020528.

3. Forbes HJ, Bhaskaran K, Thomas SL, Smeeth L, Clayton T, Mansfield K, et al. Quantification of risk factors for postherpetic neuralgia in herpes zoster patients: A cohort study. Neurology. 2016;87(1):94-102.

4. Oxman MN, Schmader KE. Editorial commentary: zoster vaccine in immunocompromised patients: time to reconsider current recommendations. Clin Infect Dis. 2014;59(7):920-2.

5. Costa E, Buxton J, Brown J, Templeton KE, Breuer J, Johannessen I. Fatal disseminated varicella zoster infection following zoster vaccination in an immunocompromised patient. BMJ Case Rep. 2016;2016.

6. Alexander KE, Tong PL, Macartney K, Beresford R, Sheppeard V, Gupta M. Live zoster vaccination in an immunocompromised patient leading to death secondary to disseminated varicella zoster virus infection. Vaccine. 2018;36(27):3890-3.

7. Zhang J, Xie F, Delzell E, Chen L, Winthrop KL, Lewis JD, et al. Association between vaccination for herpes zoster and risk of herpes zoster infection among older patients with selected immune-mediated diseases. Jama. 2012;308(1):43-9.

8. Cheetham TC, Marcy SM, Tseng HF, Sy LS, Liu IL, Bixler F, et al. Risk of Herpes Zoster and Disseminated Varicella Zoster in Patients Taking Immunosuppressant Drugs at the Time of Zoster Vaccination. Mayo Clin Proc. 2015;90(7):865-73.

For peer review only - http://bmjopen.bmj.com/site/about/guidelines.xhtml

9. Naidus E, Damon L, Schwartz BS, Breed C, Liu C. Experience with use of Zostavax((R)) in patients with hematologic malignancy and hematopoietic cell transplant recipients. Am J Hematol. 2012;87(1):123-5.

10. Tseng HF, Tartof S, Harpaz R, Luo Y, Sy LS, Hetcher RC, et al. Vaccination against zoster remains effective in older adults who later undergo chemotherapy. Clin Infect Dis. 2014;59(7):913-9.

11. Langan SM, Smeeth L, Margolis DJ, Thomas SL. Herpes zoster vaccine effectiveness against incident herpes zoster and post-herpetic neuralgia in an older US population: a cohort study. PLoS medicine. 2013;10(4):e1001420.

12. Benson CA, Andersen JW, Macatangay BJ, Mailliard RB, Rinaldo Jr CR, Read S, et al. Safety and Immunogenicity of Zoster Vaccine Live in Human Immunodeficiency Virus–Infected Adults With CD4+ Cell Counts> 200 Cells/mL Virologically Suppressed on Antiretroviral Therapy. Clinical Infectious Diseases. 2018;67(11):1712-9.

For peer review only

For peer review only - http://bmjopen.bmj.com/site/about/guidelines.xhtml

Figure legend

Cumulative uptake of zoster vaccination while immunosuppressed, by vaccination programme year. A) Stratified by maiden years of eligibility or overall eligibility. B) Stratified by routine or catch-up cohort.

For peer review only

For peer review only - http://bmjopen.bmj.com/site/about/guidelines.xhtml

For peer review only

For peer review only - http://bmjopen.bmj.com/site/about/guidelines.xhtml

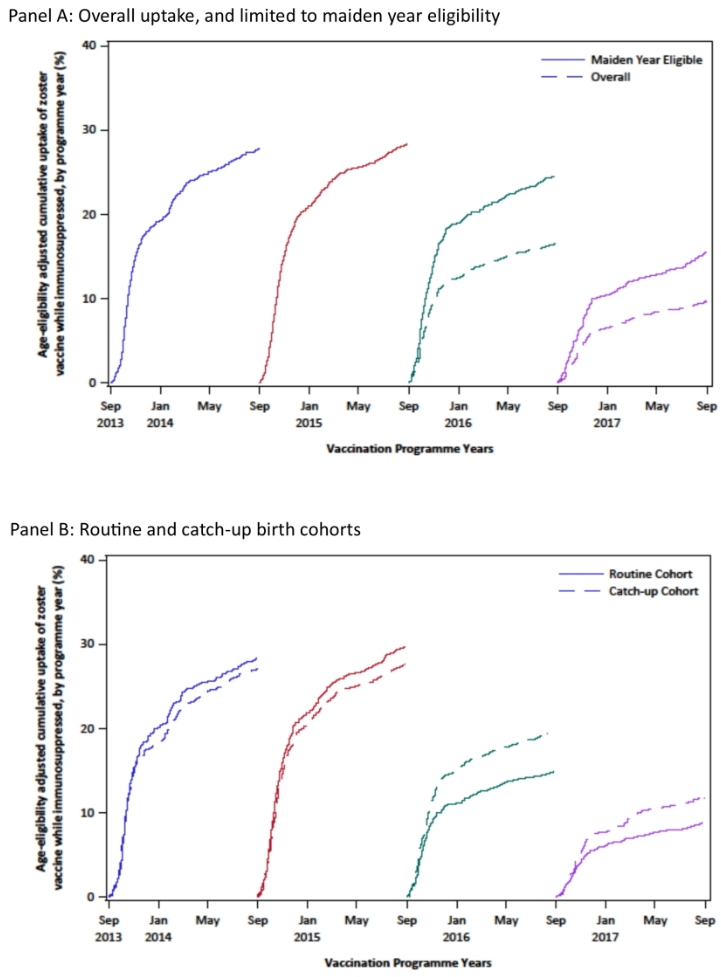

209x296mm (300 x 300 DPI)

Appendices

Appendix A1: Approach to defining age-eligible cohorts for zoster vaccination

CPRD data are anonymised and includes year of birth but not day or month. This is a challenge for identifying the age-cohorts eligible for vaccination. Age eligibility for zoster vaccination has differed for each year of the vaccination programme since its introduction, as follows:

- *2013/14*: those aged 70 or 79 years on 1st Sept 2013;
- *2014/15:* those aged 70, 78 or 79 years on 1st Sept 2014;
- *2015/16*: those aged 70, 71, 72, 78 or 79 years on 1st Sept 2015;
- *2016/17*: those aged 70, 71, 72, 73, 78 or 79 years on 1st Sept 2016.

The distribution of zoster vaccine eligibility according to year of birth is detailed in Table A1, below. We included the following age cohorts over the four years of the study to ensure that at least 67% of the cohort was age-eligible in each year:

- Study year 1: 2013/14: years of birth 1943, 1934
- Study year 2: 2014/15: years of birth 1944, 1935, 1936
- Study year 3: 2015/16: years of birth 1943*, 1944*, 1945, 1936*, 1937
- Study year 4: 2016/17: years of birth 1943*, 1944*, 1945*, 1946, 1937*, 1938

  * Included if not previously vaccinated

Age-eligibility person years at risk adjustment

The rate of vaccination while immunosuppressed was calculated by adjusting the denominator person years at risk to account for age-eligibility uncertainty relating to unknown month of birth. For example, people born in 1943 were eligible if born prior to 2nd September (Table A1), so eligibility for those born in 1943 was assumed to be 67%. Therefore, the total person years at risk of people born in 1943 was adjusted by multiplying by 0.67. This process was followed for all years with partial age-eligibility.

For cumulative uptake analysis, those born in years with partial (67%) age-eligibility contributed 0.67 to the denominator.

For peer review only

Table A1: Summary of zoster vaccine eligibility by calendar year and year of birth

Year of birth	True day/month of birth	cohort	Year 1: 09/2013-08/2014 Age at 01/09	Year 1: Eligible?	Year 2: 09/2014-08/2015 Age at 01/09	Year 2: Eligible?	Year 3: 09/2015-08/2016 Age at 01/09	Year 3: Eligible?	Year 4: 09/2016-08/2017 Age at 01/09*	Year 4: Eligible?
1943	1st Jan-1st Sep	Routine	70	Eligible	71	Ineligible	72	Eligible	73	Eligible
1943	2nd Sep-31st Dec	Routine	69	Ineligible	70	Eligible	71	Eligible	72	Eligible
1944	1st Jan-1st Sep	Routine	69	Ineligible	70	Eligible	71	Eligible	72	Eligible
1944	2nd Sep-31st Dec	Routine	68	Ineligible	69	Ineligible	70	Eligible	71	Eligible
1945	1st Jan-1st Sep	Routine	68	Ineligible	69	Ineligible	70	Eligible	71	Eligible
1945	2nd Sep-31st Dec	Routine	67	Ineligible	68	Ineligible	69	Ineligible	70	Eligible
1946	1st Jan-1st Sep	Routine	67	Ineligible	68	Ineligible	69	Ineligible	70	Eligible
1946	2nd Sep-31st Dec	Routine	66	Ineligible	67	Ineligible	68	Ineligible	69	Ineligible*
1934	1st Jan-1st Sep	Catch up	79	Eligible	80	Ineligible	81	Ineligible	82	Ineligible
1934	2nd Sep-31st Dec	Catch up	78	Ineligible	79	Eligible	80	Ineligible	81	Ineligible
1935	1st Jan-1st Sep	Catch up	78	Ineligible	79	Eligible	80	Ineligible	81	Ineligible
1935	2nd Sep-31st Dec	Catch up	77	Ineligible	78	Eligible	79	Eligible	80	Ineligible
1936	1st Jan-1st Sep	Catch up	77	Ineligible	78	Eligible	79	Eligible	80	Ineligible
1936	2nd Sep-31st Dec	Catch up	76	Ineligible	77	Ineligible	78	Eligible	79	Eligible
1937	1st Jan-1st Sep	Catch up	76	Ineligible	77	Ineligible	78	Eligible	79	Eligible
1937	2nd Sep-31st Dec	Catch up	75	Ineligible	76	Ineligible	77	Ineligible	78	Eligible
1938	1st Jan-1st Sep	Catch up	75	Ineligible	76	Ineligible	77	Ineligible	78	Eligible
1938	2nd Sep-31st Dec	Catch up	74	Ineligible	75	Ineligible	76	Ineligible	77	Ineligible*

* From April 2017, rules changed so patients became eligible on the day they turned 70yrs (routine) or 78 years (catch up); those with existing eligibility who missed vaccination could still be offered the vaccine.

For peer review only

Appendix A2: Summary of definitions of periods of immunosuppression

Immunosuppression category	Category includes	Code types used to identify immunosuppression	Time period defined as immunosuppressed following each record
Haematological malignancies	Leukaemias, lymphomas, other lymphoproliferative disorders	Read v2 codes<br>ICD-10 codes[1]	24 months
HIV/AIDS		Read v2 codes<br>ICD-10 codes[1]	Permanent
Cellular immune deficiencies	Permanent	Read v2 codes<br>ICD-10 codes[1]	Permanent
	Aplastic anaemia	Read v2 codes<br>ICD-10 codes[1]	24 months
	Other/unspecified cellular immune deficiences	Read v2 codes<br>ICD-10 codes[1]	90 days
Bone marrow transplants	Allogenic or autologous stem cell transplant	Read v2 codes<br>ICD-10 codes[1]<br>OPCS procedure codes[1]	24 months
Immunosuppressive therapies for solid organ transplant	Solid organ transplant	Read v2 codes<br>ICD-10 codes[1]<br>OPCS procedure codes[1]	Permanent
Chemotherapy or radiotherapy		Read v2 codes<br>Prescriptions<br>ICD-10 codes[1]<br>OPCS procedure codes[1]	1 year unless a record of end of therapy, in which case 6 months
Oral corticosteroids	Short term high-dose corticosteroids >40mg prednisolone per day for more than 1 week	Prescriptions	3 months
	Long term lower dose corticosteroids (>20mg prednisolone per day for more than 14 days	Prescriptions	3 months
Biological therapies		Prescriptions	3 months before first ever prescription[2] and 12 months following each prescription
Other immunosuppressant medications	Methotrexate >25mg per week; Azathioprine >3.0mg/kg/day; 6-mercaptopurine >1.5mg/kg/day; Corticosteroid injections; other DMARDS; other immunosuppressant medications	Prescriptions	3 months before first ever prescription[2] and 3 months following each prescription

1. For patients with available linkage to Hospital Episode Statistics (HES)
2. To reflect standard practice of initiation in secondary care.

For peer review only

STROBE Statement—Checklist of items that should be included in reports of ***cohort studies***

	Item No	Recommendation	Page
**Title and abstract**	1	(*a*) Indicate the study's design with a commonly used term in the title or the abstract	1
		(*b*) Provide in the abstract an informative and balanced summary of what was done and what was found	2
**Introduction**			
Background/rationale	2	Explain the scientific background and rationale for the investigation being reported	4
Objectives	3	State specific objectives, including any prespecified hypotheses	4
**Methods**			
Study design	4	Present key elements of study design early in the paper	6
Setting	5	Describe the setting, locations, and relevant dates, including periods of recruitment, exposure, follow-up, and data collection	5-6
Participants	6	(*a*) Give the eligibility criteria, and the sources and methods of selection of participants. Describe methods of follow-up	5
		(*b*) For matched studies, give matching criteria and number of exposed and unexposed	N/A
Variables	7	Clearly define all outcomes, exposures, predictors, potential confounders, and effect modifiers. Give diagnostic criteria, if applicable	5-6
Data sources/ measurement	8*	For each variable of interest, give sources of data and details of methods of assessment (measurement). Describe comparability of assessment methods if there is more than one group	*5-6*
Bias	9	Describe any efforts to address potential sources of bias	6
Study size	10	Explain how the study size was arrived at	5
Quantitative variables	11	Explain how quantitative variables were handled in the analyses. If applicable, describe which groupings were chosen and why	5-6
Statistical methods	12	(*a*) Describe all statistical methods, including those used to control for confounding	6
		(*b*) Describe any methods used to examine subgroups and interactions	N/A
		(*c*) Explain how missing data were addressed	5
		(*d*) If applicable, explain how loss to follow-up was addressed	N/A
		(*e*) Describe any sensitivity analyses	6
**Results**			
Participants	13*	(a) Report numbers of individuals at each stage of study—eg numbers potentially eligible, examined for eligibility, confirmed eligible, included in the study, completing follow-up, and analysed	8
		(b) Give reasons for non-participation at each stage	8
		(c) Consider use of a flow diagram	N/A

For peer review only - http://bmjopen.bmj.com/site/about/guidelines.xhtml

Descriptive data	14*	(a) Give characteristics of study participants (eg demographic, clinical, social) and information on exposures and potential confounders	8-9
		(b) Indicate number of participants with missing data for each variable of interest	N/A
		(c) Summarise follow-up time (eg, average and total amount)	8
Outcome data	15*	Report numbers of outcome events or summary measures over time	8-9
Main results	16	(*a*) Give unadjusted estimates and, if applicable, confounder-adjusted estimates and their precision (eg, 95% confidence interval). Make clear which confounders were adjusted for and why they were included	8-9
		(*b*) Report category boundaries when continuous variables were categorized	N/A
		(*c*) If relevant, consider translating estimates of relative risk into absolute risk for a meaningful time period	8-9
Other analyses	17	Report other analyses done—eg analyses of subgroups and interactions, and sensitivity analyses	8-9
**Discussion**			
Key results	18	Summarise key results with reference to study objectives	10
Limitations	19	Discuss limitations of the study, taking into account sources of potential bias or imprecision. Discuss both direction and magnitude of any potential bias	10-11
Interpretation	20	Give a cautious overall interpretation of results considering objectives, limitations, multiplicity of analyses, results from similar studies, and other relevant evidence	11
Generalisability	21	Discuss the generalisability (external validity) of the study results	11
**Other information**			
Funding	22	Give the source of funding and the role of the funders for the present study and, if applicable, for the original study on which the present article is based	12

*Give information separately for exposed and unexposed groups.

Note: An Explanation and Elaboration article discusses each checklist item and gives methodological background and published examples of transparent reporting. The STROBE checklist is best used in conjunction with this article (freely available on the Web sites of PLoS Medicine at http://www.plosmedicine.org/, Annals of Internal Medicine at http://www.annals.org/, and Epidemiology at http://www.epidem.com/). Information on the STROBE Initiative is available at http://www.strobe-statement.org.

For peer review only

BMJ Open

Safety of inadvertent administration of live zoster vaccine to immunosuppressed individuals in a UK-based observational cohort analysis

Journal:	*BMJ Open*
Manuscript ID	bmjopen-2019-034886.R1
Article Type:	Original research
Date Submitted by the Author:	17-Dec-2019
Complete List of Authors:	Grint, Daniel; London School of Hygiene and Tropical Medicine, Infectious Disease Epidemiology<br>McDonald, Helen; London School of Hygiene and Tropical Medicine, Infectious Disease Epidemiology<br>Walker, Jemma; London School of Hygiene and Tropical Medicine, Faculty of Epidemiology and Population Health; Public Health England, Statistics, Modelling and Economics Department<br>Amirthalingam, Gayatri; Public Health England, Immunisation and Countermeasures Division<br>Andrews, Nick; Public Health England, Statistics, Modelling and Economics Department; London School of Hygiene and Tropical Medicine, Infectious Disease Epidemiology<br>Thomas, Sara; London School of Hygiene and Tropical Medicine, Infectious Disease Epidemiology
**Primary Subject Heading**:	Public health
Secondary Subject Heading:	Epidemiology
Keywords:	Geriatric dermatology < DERMATOLOGY, IMMUNOLOGY, Public health < INFECTIOUS DISEASES, PUBLIC HEALTH, Adverse events < THERAPEUTICS, Infectious diseases & infestations < DERMATOLOGY

SCHOLARONE™
Manuscripts

I, the Submitting Author has the right to grant and does grant on behalf of all authors of the Work (as defined in the below author licence), an exclusive licence and/or a non-exclusive licence for contributions from authors who are: i) UK Crown employees; ii) where BMJ has agreed a CC-BY licence shall apply, and/or iii) in accordance with the terms applicable for US Federal Government officers or employees acting as part of their official duties; on a worldwide, perpetual, irrevocable, royalty-free basis to BMJ Publishing Group Ltd ("BMJ") its licensees and where the relevant Journal is co-owned by BMJ to the co-owners of the Journal, to publish the Work in this journal and any other BMJ products and to exploit all rights, as set out in our licence.

The Submitting Author accepts and understands that any supply made under these terms is made by BMJ to the Submitting Author unless you are acting as an employee on behalf of your employer or a postgraduate student of an affiliated institution which is paying any applicable article publishing charge ("APC") for Open Access articles. Where the Submitting Author wishes to make the Work available on an Open Access basis (and intends to pay the relevant APC), the terms of reuse of such Open Access shall be governed by a Creative Commons licence – details of these licences and which Creative Commons licence will apply to this Work are set out in our licence referred to above.

Other than as permitted in any relevant BMJ Author's Self Archiving Policies, I confirm this Work has not been accepted for publication elsewhere, is not being considered for publication elsewhere and does not duplicate material already published. I confirm all authors consent to publication of this Work and authorise the granting of this licence.

For peer review only

Safety of inadvertent administration of live zoster vaccine to immunosuppressed individuals in a UK-based observational cohort analysis

For peer review only

Authors: Daniel J Grint (0000-0001-8001-6544),[1] Helen I McDonald (0000-0003-0576-2015),[1] Jemma L Walker,[1,2] Gayatri Amirthalingam,[3] Nick Andrews,[1,2] Sara L Thomas.[1]

1. Department of Infectious Disease Epidemiology, London School of Hygiene & Tropical Medicine, London, UK

Daniel J Grint assistant professor, Helen I McDonald clinical research fellow, Jemma L Walker research fellow, Nick Andrews honorary professor, Sara L Thomas professor (retired).

2. Statistics, Modelling and Economics Department, Public Health England, London, UK

Jemma L Walker statistician, Nick Andrews senior statistician.

3. Immunisation and Countermeasures Division, National Infection Service, Public Health England, London, UK

Gayatri Amirthalingam consultant epidemiologist.

Correspondence to: Helen McDonald, London School of Hygiene & Tropical Medicine, Keppel Street, London, WC1E 7HT. Helen.mcdonald@lshtm.ac.uk Tel: 020 7612 7921 Fax: N/A

Article type: Research

For peer review only - http://bmjopen.bmj.com/site/about/guidelines.xhtml

Abstract

Objectives:

To investigate the safety of live attenuated varicella zoster vaccination when administered to immunosuppressed individuals.

Design:

Prospective observational cohort study.

Setting:

The study used anonymised data from the Clinical Practice Research Datalink (CPRD), comprising a representative sample of routinely collected primary care data in England between 2013 and 2017, and linked Hospital Episode Statistics (HES) data.

Participants:

168,767 individuals age-eligible for varicella zoster vaccination registered at a general practice in England contributing data to CPRD.

Main outcome measures:

Electronic health records of conditions indicating immunosuppression, administration of zoster vaccination, diagnoses of specific zoster-related disease and non-specific rash compatible with zoster disease.

Results:

Between 1st September 2013 and 31st August 2017, a period of immunosuppression was identified for 9,093/168,767 (5·4%; 95% CI: 5·3-5·5%) individuals age-eligible for zoster vaccination. The overall rate of vaccination while immunosuppressed was 1,742/5,251 (33·2 per 100 adjusted person years at risk; 95% CI: 31·9-34·5). Follow-up of the 1,742 individuals who were inadvertently vaccinated while immunosuppressed identified only two zoster cases of within 8 weeks of vaccination (0·1%; 95% CI: 0·01-0·4%), both primary care diagnoses of "shingles", and neither with a related hospital admission.

Conclusions:

Despite evidence of inadvertent vaccination of immunosuppressed individuals with live zoster vaccination, there is a lack of evidence of severe consequences including hospitalisation. This should reassure primary care staff and encourage vaccination of mildly immunosuppressed individuals who

For peer review only - http://bmjopen.bmj.com/site/about/guidelines.xhtml

do not meet current thresholds for contraindication. These findings support a review of the extent to which live zoster vaccination is contraindicated among the immunosuppressed.

For peer review only

For peer review only - http://bmjopen.bmj.com/site/about/guidelines.xhtml

Strengths and limitations

- This study investigated the safety of live zoster vaccination during immunosuppression in a large national cohort using electronic health records.
- It is the first study to cover the full profile of causes of immunosuppression listed as contraindications to vaccination in UK national guidance, ascertained from multiple primary and secondary care sources.
- Both primary and secondary care records were used for thorough ascertainment of zoster cases, including a sensitivity analysis ascertaining non-specific rash or encephalitis of unspecified aetiology.
- Vaccination rates were analysed using only year of birth for age-eligibility, and so the denominator was adjusted for birth cohorts with partial eligibility.
- Immunosuppression was not distinguished according to severity, but clinicians may have vaccinated selectively and caution would be needed in applying these findings outside of current vaccination practice.

For peer review only

Introduction

Herpes zoster (shingles) is a common and painful disease caused by reactivation of varicella zoster virus (VZV), with debilitating complications including post-herpetic neuralgia. Live-attenuated zoster vaccine was introduced for immunocompetent adults aged 70-79 years in England in 2013, delivered in primary care.(1) The herpes zoster vaccination programme in England was found to have a population impact equivalent to approximately 17,000 fewer episodes of herpes zoster and 3,300 fewer episodes of postherpetic neuralgia among 5·5 million eligible individuals in the first 3 years of the programme.(2)

Immunosuppression is associated with a high burden of zoster and its complications,(3, 4) and there have been calls to consider vaccination for this population.(5) However, live zoster vaccine is currently contraindicated in immunosuppression.(1) High-profile case reports of fatal vaccine-related disease among severely immunosuppressed individuals have caused concern and may have contributed to declining vaccine coverage.(6, 7) Understanding the safety of live vaccination during immunosuppression is important to support guidance on use of the vaccine, to ensure that individuals who can safely benefit from the vaccine are enabled to do so.

A new vaccine which is recombinant rather than containing live virus could offer a safer alternative for immunosuppressed individuals without the risk of vaccine-related zoster, and has been found to be effective among patients with autologous hematopoietic stem cell transplantation (HSCT).(8) However, supplies are currently unable to meet global demands. The Joint Committee on Vaccination and Immunisation has recommended use of the recombinant vaccine for individuals with immunosuppression. Understanding the safety of live vaccination for typical causes of immunosuppression will be important to prioritise use of limited supplies of the new recombinant vaccine. When recombinant vaccine is used in cases for which live vaccine is contraindicated, this may introduce potential for confusion and it will be even more important to understand the consequences of inadvertent live vaccination during immunosuppression.

For peer review only - http://bmjopen.bmj.com/site/about/guidelines.xhtml

This study aimed to investigate the frequency and consequences of live zoster vaccination during immunosuppression among a large UK cohort from 2013 to 2017.

For peer review only - http://bmjopen.bmj.com/site/about/guidelines.xhtml

Methods

Data source

This study used anonymised data from the Clinical Practice Research Datalink (CPRD). The data include information on year of birth, medical diagnoses (version 2 Read codes), prescriptions and vaccinations. For 60% of individuals, records are pre-linked to anonymised hospitalisation data (Hospital Episode Statistics, HES). HES-linked data for inpatient admissions (International Classification of Disease, ICD-10 codes) and procedures (OPCS-4 Classification of Procedures codes) were used to supplement identification of immunosuppressed individuals and zoster disease.

Study population

Immunosuppressed individuals age-eligible for zoster vaccination, active in CPRD from September 2013 to August 2017 and registered with a CPRD practice for at least a year before study entry, were included.

Age eligibility for zoster vaccination has differed each year since the vaccination introduction. As month of birth was not available, individuals born in years for which ≥67% of the population would have been eligible for vaccination were included (Appendix A1). Birth cohorts were defined as 'maiden cohorts' in the first year for which they were age-eligible for vaccination.

Periods of immunosuppression were identified using Read codes and prescription records from CPRD, plus ICD-10 codes and OPCS codes in linked HES data using algorithms previously described.(9) Immunosuppression was defined based on contraindications to live zoster vaccination described in national guidance.(1) The time periods assigned to each immunosuppressing condition or medication type, and dose thresholds for relevant medications, are described in Appendix A2. For prescription records missing dose, the median was imputed according to category of age and sex, in line with previous zoster studies.(4)

Vaccination status and vaccine-related varicella zoster disease

For peer review only - http://bmjopen.bmj.com/site/about/guidelines.xhtml

Individuals were followed to the first positive record of zoster vaccination. If this indicated that the vaccine was delivered by another health care provider the individual was excluded from the cohort, as timing of vaccination could not be determined (N=29).

Evidence of varicella zoster disease in primary or secondary care records was assessed during the 8 weeks following a vaccination given while immunosuppressed. For the primary analysis, only specific diagnoses of VZV disease were included. Sensitivity analysis also included any rash that was unspecified or compatible with VZV, and acute encephalitis of unspecified aetiology. For individuals with HES-linkage, any diagnosis recorded within 8 weeks following vaccination was used to supplement identification of varicella-zoster disease. Codelists are available at https://datacompass.lshtm.ac.uk/1336/.

Statistical methods

An open prospective cohort study design was used whereby individuals exited and re-entered the cohort according to time-varying immunosuppression status. Follow-up started at 1st September of the study year in which the individual was age-eligible for vaccination and ended at the earliest of death, date of transfer out of practice, practice last collection date, elapsed age-eligibility for zoster vaccination, resolution of immunosuppression, or 31st August 2017.

The vaccination rate was calculated by total person years at risk (PYAR) while immunosuppressed with adjustment to account for age-eligibility uncertainty from unknown month of birth (Appendix A1). Cumulative uptake of zoster vaccine was computed stratified by treatment cohort and programme year.

The number of vaccinated immunosuppressed individuals who developed VZV disease in the subsequent eight weeks was described. In sensitivity analysis, disease in the first week following vaccination was excluded.

Statistical analysis was performed using STATA version 14·2 and SAS version 9.4.

For peer review only - http://bmjopen.bmj.com/site/about/guidelines.xhtml

Ethics approval

The study was approved by the Independent Scientific Advisory Group of the CPRD (ISAC reference 18_218A) and the London School of Hygiene and Tropical Medicine Ethics Committee (reference 16298). The amended ISAC protocol was made available to reviewers.

Patient and public involvement

Patients and the public were not involved in the design or planning of the study.

For peer review only

For peer review only - http://bmjopen.bmj.com/site/about/guidelines.xhtml

Results

Between 1st September 2013 and 31st August 2017 data were available for 168,767 individuals age-eligible for vaccination, of whom 89,416 (53·0%) were female and 76,337 (45·2%) in the catch-up cohort. A period of immunosuppression while age-eligible for vaccination was identified for 9,093/168,767 (5·4%; 95% CI: 5·3-5·5%).

1,742 individuals were vaccinated during a period of immunosuppression. Adjusting person years at risk while immunosuppressed to account for age-eligibility uncertainty, the overall rate of vaccination during immunosuppression was 1,742/5,251 (33·2 per 100 adjusted person years at risk; 95% CI: 31·9-34·5). Figure 1 shows the cumulative uptake of zoster vaccine by programme year overall, in maiden years of eligibility and by cohort. Cumulative uptake was higher in programme years 3 and 4 when restricted to maiden years of eligibility. Cumulative uptake was highest in programme years 1 and 2 for both the routine and catch-up cohorts.

Among those vaccinated while immunosuppressed the most common underlying cause was chemotherapy (55·3%), followed by other immunosuppressant therapies including biologics (13·2%), multiple indications (11·4%), and steroid drug use (11·3%). 47 (2·7%) had a permanent cause of immunosuppression. 368 (21·1%) were immunosuppressed for the duration of follow-up; median follow-up 32·2 months (IQR: 19·7-48·0). Vaccination took place during the final four weeks of a defined period of immunosuppression for 138/1,742 (7·9%).

In the eight weeks following vaccination 2/1,742 (0·1%; 95% CI: 0·01-0·4%) had a diagnosis of shingles recorded in primary care. Both individuals had HES-linkage available, however, neither had a related hospital admission. One of these cases occurred within 7 days of vaccination.

Using a broader definition including non-specific rash or encephalitis identified a further 23 possible cases of VZV disease (in total 25/1,742 (1·4%; 95% CI: 0·9-2·1%)). All of these were instances of

For peer review only - http://bmjopen.bmj.com/site/about/guidelines.xhtml

unspecified rash in primary care, and there were no cases of encephalitis. In total, 22/25 (88%) had HES-linkage available, however, none had a related hospital admission recorded. Five of the broader definition cases occurred within 7 days of vaccine administration.

Chemotherapy was the cause of immunosuppression for the majority of cases who developed specific or non-specific potentially vaccine-related disease (15/25 (60%)). The remaining cases included immunosuppression by oral steroid use, other immunosuppressant medications, leukaemia, and organ transplant.

For peer review only

For peer review only - http://bmjopen.bmj.com/site/about/guidelines.xhtml

Discussion

This is the first study to investigate the safety of live zoster vaccination across the range of contraindicating immunosuppressive conditions. Our study identified 1,742 individuals vaccinated while immunosuppressed and two subsequent cases of shingles, with no related hospitalisations.

A key strength of this study is the thorough ascertainment of both immunosuppression and VZV disease using linked primary and secondary care data for a large, representative cohort with a range of immunosuppressive conditions.

The study has limitations. A key limitation is that month of birth was not available for precise identification of age-eligibility. If immunosuppressed individuals in a birth cohort with 67% eligibility were vaccinated while not age-eligible, rates of vaccination in immunosuppression would be overestimated. There also remains uncertainty in defining time-periods of immunosuppression and in imputing missing dose data for medications, which may result in under- or over-ascertainment of immunosuppression.

It is possible that zoster disease may have been under-ascertained, either because patients did not attend healthcare or due to non-specific coding of zoster disease. A US study reported that 95% of patients aged over 60 years had attended healthcare when they experienced zoster disease,(10) and this might be expected to be higher among immunosuppressed individuals in a setting with universal healthcare. This study used both primary care and secondary care records to ascertain zoster cases, an approach which has previously been found to generate plausible estimates of zoster incidence among the older general population.(11) The sensitivity analysis was also designed to ascertain possible cases of zoster which may have been coded non-specifically as rash or encephalitis. Conversely, as this population has a high baseline risk of naturally occurring shingles, we may have over-estimated the risk attributable to vaccination, particularly when including cases within 7 days of vaccination.

For peer review only - http://bmjopen.bmj.com/site/about/guidelines.xhtml

Finally, while our definitions of immunosuppression followed national guidance, we could not replace clinical judgement on severity or timing of immunosuppression, and the study was not powered to assess safety according to type of immunosuppression.(1) Clinicians may have selectively, rather than inadvertently, vaccinated individuals at lower risk of vaccine-related disease, resulting in a 'healthy vaccinee' effect, and caution would be needed in generalising these findings outside of current practice in the context of guidance on contraindications. However, the most frequent contraindication was chemotherapy, and vaccinations did not occur disproportionately towards the end of a period of immunosuppression, suggesting that vaccinations were not all at the margins of the guidance.

Our safety findings are consistent with previous studies of live zoster vaccination among patients with selected immunosuppressive conditions.(12-14) Studies showing that VZV-specific immunity may persist or even be boosted by vaccination during cell-mediated immunosuppression,(15-17) further support the plausibility of residual immunity against vaccine-related disease despite immunosuppression.

Rates of zoster vaccination during immunosuppression were high, and similar in routine and catch-up cohorts. To our knowledge, this is the first study to calculate rates of live zoster vaccination across this range of immunosuppressing conditions. A zoster vaccine effectiveness study of Medicare beneficiaries in the US included 140,925 individuals with a diagnosis of leukaemia, lymphoma or HIV, of whom 4,469 (3.2%) were vaccinated while immunosuppressed, comparable to the overall study vaccine uptake (29,785/766,330; 3.9%), suggesting that live zoster vaccination despite immunosuppression is not unique to the UK setting.(16) In our study, analysis restricted to maiden years of eligibility suggests that the apparent decline in vaccination rates after year 2 is partly a cohort effect, whereby people who were unvaccinated despite previous eligibility were less likely to be vaccinated subsequently. This could be due to an initial decision not to vaccinate continuing over subsequent years of eligibility, or a greater focus on vaccination for newly eligible patients. Increasingly detailed guidance over time may also have helped reduce inadvertent vaccination.

For peer review only - http://bmjopen.bmj.com/site/about/guidelines.xhtml

Among this large cohort with a range of immunosuppressing conditions we found no evidence of severe disease following live zoster vaccination while immunosuppressed. This should reassure clinicians, and encourage vaccination of mildly immunosuppressed individuals who do not meet current thresholds for contraindication, especially in the current context of declining uptake of the national programme.(18) These findings support a review of the extent to which live zoster vaccination is contraindicated among the immunosuppressed population. Further research is needed to identify any patient groups for whom recombinant zoster vaccine should be prioritised once stocks become available in the UK.

For peer review only

For peer review only - http://bmjopen.bmj.com/site/about/guidelines.xhtml

Role of the funding source

The research was funded by the National Institute for Health Research (NIHR) Health Protection Research Unit (HPRU) in Immunisation at the London School of Hygiene and Tropical Medicine in partnership with Public Health England (PHE). The funders had no role in the study design, collection analysis or interpretation of data, writing of the report nor the decision to submit the article for publication. The views expressed are those of the authors and not necessarily those of the NHS, the NIHR, the Department of Health and Social Care, or PHE.

Contributor and guarantor information:

SLT had the idea for the study. SLT, JLW, GA and NA obtained the data. All authors contributed to the design of the study. DJG analysed the data. All authors contributed to the interpretation of results. DJG and HIM drafted the manuscript and all authors revised it critically. All authors approved the final version to be published and agree to be accountable for all aspects of the work. DJG is the guarantor. The corresponding author attests that all listed authors meet authorship criteria and that no others meeting the criteria have been omitted.

Transparency declaration: DJG affirms that the manuscript is an honest, accurate, and transparent account of the study being reported; that no important aspects of the study have been omitted; and that any discrepancies from the study as originally planned (and, if relevant, registered) have been explained.

Competing interests declaration: All authors have completed the ICMJE uniform disclosure form at www.icmje.org/coi_disclosure.pdf and declare: DJG, JLW, NA, and SLT had financial support from the National Institute for Health Research (NIHR) Health Protection Research Unit (HPRU) in Immunisation for the submitted work; the Public Health England Immunisation Department has provided vaccine manufacturers with post-marketing surveillance reports on pneumococcal and meningococcal infection which the companies are required to submit to the UK Licensing Authority in compliance with their Risk Management Strategy, and a cost recovery charge is made for these

For peer review only - http://bmjopen.bmj.com/site/about/guidelines.xhtml

reports; no other financial relationships with any organisations that might have an interest in the submitted work in the previous three years; no other relationships or activities that could appear to have influenced the submitted work.

Licence for publication

The Corresponding Author has the right to grant on behalf of all authors and does grant on behalf of all authors, a worldwide licence to the Publishers and its licensees in perpetuity, in all forms, formats and media (whether known now or created in the future), to i) publish, reproduce, distribute, display and store the Contribution, ii) translate the Contribution into other languages, create adaptations, reprints, include within collections and create summaries, extracts and/or, abstracts of the Contribution, iii) create any other derivative work(s) based on the Contribution, iv) to exploit all subsidiary rights in the Contribution, v) the inclusion of electronic links from the Contribution to third party material where-ever it may be located; and, vi) licence any third party to do any or all of the above.

Data sharing statement

These data were obtained from the Clinical Practice Research Datalink, provided by the UK Medicines and Healthcare products Regulatory Agency. The authors' licence for using these data does not allow sharing of raw data with third parties. Information about access to Clinical Practice Research Datalink data is available here: https://www.cprd.com/research-applications

Codelists for this study are available at https://datacompass.lshtm.ac.uk/1336/.

For peer review only

References

1. Public Health England. Immunisation against Infectious Disease (the Green Book). Chapter 28a: Shingles (herpes zoster) 2016. Available from: https://assets.publishing.service.gov.uk/government/uploads/system/uploads/attachment_data/file/503773/2905109_Green_Book_Chapter_28a_v3_0W.PDF
2. Amirthalingam G, Andrews N, Keel P, Mullett D, Correa A, de Lusignan S, et al. Evaluation of the effect of the herpes zoster vaccination programme 3 years after its introduction in England: a population-based study. The lancet Public Health. 2018;3(2):e82-e90.
3. Yanni EA, Ferreira G, Guennec M, El Hahi Y, El Ghachi A, Haguinet F, et al. Burden of herpes zoster in 16 selected immunocompromised populations in England: a cohort study in the Clinical Practice Research Datalink 2000-2012. BMJ open. 2018;8(6):e020528.
4. Forbes HJ, Bhaskaran K, Thomas SL, Smeeth L, Clayton T, Mansfield K, et al. Quantification of risk factors for postherpetic neuralgia in herpes zoster patients: A cohort study. Neurology. 2016;87(1):94-102.
5. Oxman MN, Schmader KE. Editorial commentary: zoster vaccine in immunocompromised patients: time to reconsider current recommendations. Clin Infect Dis. 2014;59(7):920-2.
6. Costa E, Buxton J, Brown J, Templeton KE, Breuer J, Johannessen I. Fatal disseminated varicella zoster infection following zoster vaccination in an immunocompromised patient. BMJ Case Rep. 2016;2016.
7. Alexander KE, Tong PL, Macartney K, Beresford R, Sheppeard V, Gupta M. Live zoster vaccination in an immunocompromised patient leading to death secondary to disseminated varicella zoster virus infection. Vaccine. 2018;36(27):3890-3.
8. Bastidas A, de la Serna J, El Idrissi M, Oostvogels L, Quittet P, Lopez-Jimenez J, et al. Effect of Recombinant Zoster Vaccine on Incidence of Herpes Zoster After Autologous Stem Cell Transplantation: A Randomized Clinical Trial. JAMA. 2019;322(2):123-33.
9. Walker JL, Andrews NJ, Amirthalingam G, Forbes H, Langan SM, Thomas SL. Effectiveness of herpes zoster vaccination in an older United Kingdom population. Vaccine. 2018;36(17):2371-7.
10. Lu PJ, Euler GL, Jumaan AO, Harpaz R. Herpes zoster vaccination among adults aged 60 years or older in the United States, 2007: uptake of the first new vaccine to target seniors. Vaccine. 2009;27(6):882-7.
11. Jain A, van Hoek AJ, Walker JL, Forbes HJ, Langan SM, Root A, et al. Inequalities in zoster disease burden: a population-based cohort study to identify social determinants using linked data from the U.K. Clinical Practice Research Datalink. The British journal of dermatology. 2018;178(6):1324-30.
12. Zhang J, Xie F, Delzell E, Chen L, Winthrop KL, Lewis JD, et al. Association between vaccination for herpes zoster and risk of herpes zoster infection among older patients with selected immune-mediated diseases. Jama. 2012;308(1):43-9.
13. Cheetham TC, Marcy SM, Tseng HF, Sy LS, Liu IL, Bixler F, et al. Risk of Herpes Zoster and Disseminated Varicella Zoster in Patients Taking Immunosuppressant Drugs at the Time of Zoster Vaccination. Mayo Clin Proc. 2015;90(7):865-73.
14. Naidus E, Damon L, Schwartz BS, Breed C, Liu C. Experience with use of Zostavax((R)) in patients with hematologic malignancy and hematopoietic cell transplant recipients. Am J Hematol. 2012;87(1):123-5.
15. Tseng HF, Tartof S, Harpaz R, Luo Y, Sy LS, Hetcher RC, et al. Vaccination against zoster remains effective in older adults who later undergo chemotherapy. Clin Infect Dis. 2014;59(7):913-9.
16. Langan SM, Smeeth L, Margolis DJ, Thomas SL. Herpes zoster vaccine effectiveness against incident herpes zoster and post-herpetic neuralgia in an older US population: a cohort study. PLoS medicine. 2013;10(4):e1001420.

For peer review only - http://bmjopen.bmj.com/site/about/guidelines.xhtml

17. Benson CA, Andersen JW, Macatangay BJ, Mailliard RB, Rinaldo Jr CR, Read S, et al. Safety and Immunogenicity of Zoster Vaccine Live in Human Immunodeficiency Virus–Infected Adults With CD4+ Cell Counts> 200 Cells/mL Virologically Suppressed on Antiretroviral Therapy. Clinical Infectious Diseases. 2018;67(11):1712-9.
18. Public Health England. Herpes zoster (shingles) immunisation programme 2017 to 2018: evaluation report Health Protection Report: PHE Publications gateway number:2018660; 2018.

For peer review only

For peer review only - http://bmjopen.bmj.com/site/about/guidelines.xhtml

Figure legend

Cumulative uptake of zoster vaccination while immunosuppressed, by vaccination programme year.

A) Stratified by maiden years of eligibility or overall eligibility. B) Stratified by routine or catch-up cohort.

For peer review only

For peer review only - http://bmjopen.bmj.com/site/about/guidelines.xhtml

For peer review only

For peer review only - http://bmjopen.bmj.com/site/about/guidelines.xhtml

Panel A: Overall uptake, and limited to maiden year eligibility

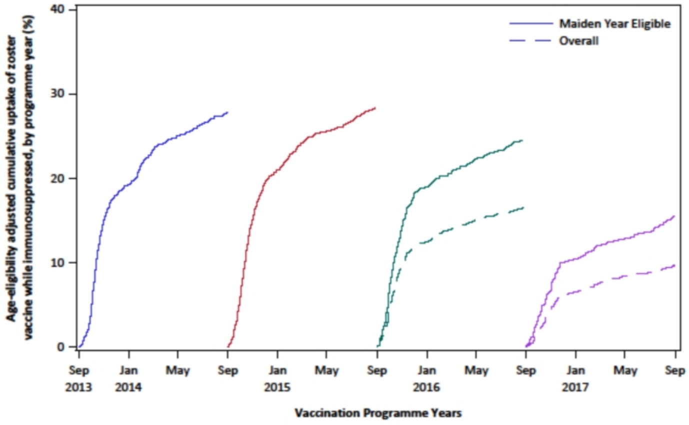

Panel B: Routine and catch-up birth cohorts

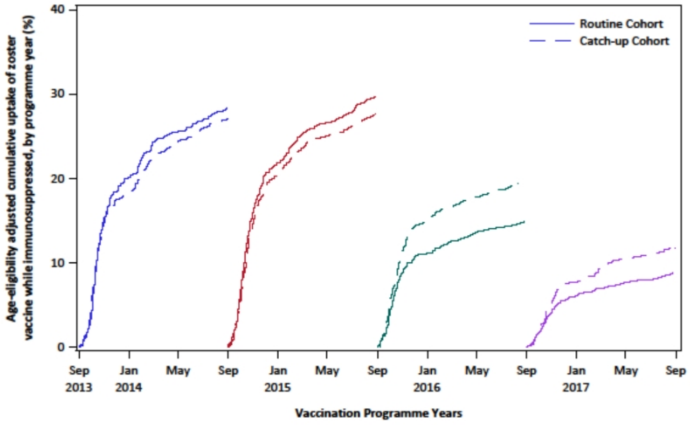

209x296mm (300 x 300 DPI)

Appendices

Appendix A1: Approach to defining age-eligible cohorts for zoster vaccination

CPRD data are anonymised and includes year of birth but not day or month. This is a challenge for identifying the age-cohorts eligible for vaccination. Age eligibility for zoster vaccination has differed for each year of the vaccination programme since its introduction, as follows:

- *2013/14*: those aged 70 or 79 years on 1st Sept 2013;
- *2014/15:* those aged 70, 78 or 79 years on 1st Sept 2014;
- *2015/16*: those aged 70, 71, 72, 78 or 79 years on 1st Sept 2015;
- *2016/17*: those aged 70, 71, 72, 73, 78 or 79 years on 1st Sept 2016.

The distribution of zoster vaccine eligibility according to year of birth is detailed in Table A1, below. We included the following age cohorts over the four years of the study to ensure that at least 67% of the cohort was age-eligible in each year:

- Study year 1: 2013/14: years of birth 1943, 1934
- Study year 2: 2014/15: years of birth 1944, 1935, 1936
- Study year 3: 2015/16: years of birth 1943*, 1944*, 1945, 1936*, 1937
- Study year 4: 2016/17: years of birth 1943*, 1944*, 1945*, 1946, 1937*, 1938

  * Included if not previously vaccinated

Age-eligibility person years at risk adjustment

The rate of vaccination while immunosuppressed was calculated by adjusting the denominator person years at risk to account for age-eligibility uncertainty relating to unknown month of birth. For example, people born in 1943 were eligible if born prior to 2nd September (Table A1), so eligibility for those born in 1943 was assumed to be 67%. Therefore, the total person years at risk of people born in 1943 was adjusted by multiplying by 0.67. This process was followed for all years with partial age-eligibility.

For cumulative uptake analysis, those born in years with partial (67%) age-eligibility contributed 0.67 to the denominator.

For peer review only

Table A1: Summary of zoster vaccine eligibility by calendar year and year of birth

			Year 1: 09/2013-08/2014		Year 2: 09/2014-08/2015		Year 3: 09/2015-08/2016		Year 4: 09/2016-08/2017	
**Year of birth**	**True day/month of birth**	**cohort**	**Age at 01/09**	**Eligible?**	**Age at 01/09**	**Eligible?**	**Age at 01/09**	**Eligible?**	**Age at 01/09***	**Eligible?**
1943	1st Jan-1st Sep	Routine	70	Eligible	71	Ineligible	72	Eligible	73	Eligible
1943	2nd Sep-31st Dec	Routine	69	Ineligible	70	Eligible	71	Eligible	72	Eligible
1944	1st Jan-1st Sep	Routine	69	Ineligible	70	Eligible	71	Eligible	72	Eligible
1944	2nd Sep-31st Dec	Routine	68	Ineligible	69	Ineligible	70	Eligible	71	Eligible
1945	1st Jan-1st Sep	Routine	68	Ineligible	69	Ineligible	70	Eligible	71	Eligible
1945	2nd Sep-31st Dec	Routine	67	Ineligible	68	Ineligible	69	Ineligible	70	Eligible
1946	1st Jan-1st Sep	Routine	67	Ineligible	68	Ineligible	69	Ineligible	70	Eligible
1946	2nd Sep-31st Dec	Routine	66	Ineligible	67	Ineligible	68	Ineligible	69	Ineligible*
1934	1st Jan-1st Sep	Catch up	79	Eligible	80	Ineligible	81	Ineligible	82	Ineligible
1934	2nd Sep-31st Dec	Catch up	78	Ineligible	79	Eligible	80	Ineligible	81	Ineligible
1935	1st Jan-1st Sep	Catch up	78	Ineligible	79	Eligible	80	Ineligible	81	Ineligible
1935	2nd Sep-31st Dec	Catch up	77	Ineligible	78	Eligible	79	Eligible	80	Ineligible
1936	1st Jan-1st Sep	Catch up	77	Ineligible	78	Eligible	79	Eligible	80	Ineligible
1936	2nd Sep-31st Dec	Catch up	76	Ineligible	77	Ineligible	78	Eligible	79	Eligible
1937	1st Jan-1st Sep	Catch up	76	Ineligible	77	Ineligible	78	Eligible	79	Eligible
1937	2nd Sep-31st Dec	Catch up	75	Ineligible	76	Ineligible	77	Ineligible	78	Eligible
1938	1st Jan-1st Sep	Catch up	75	Ineligible	76	Ineligible	77	Ineligible	78	Eligible
1938	2nd Sep-31st Dec	Catch up	74	Ineligible	75	Ineligible	76	Ineligible	77	Ineligible*

* From April 2017, rules changed so patients became eligible on the day they turned 70yrs (routine) or 78 years (catch up); those with existing eligibility who missed vaccination could still be offered the vaccine.

For peer review only

Appendix A2: Summary of definitions of periods of immunosuppression

Immunosuppression category	Category includes	Code types used to identify immunosuppression	Time period defined as immunosuppressed following each record
Haematological malignancies	Leukaemias, lymphomas, other lymphoproliferative disorders	Read v2 codes<br>ICD-10 codes[1]	24 months
HIV/AIDS		Read v2 codes<br>ICD-10 codes[1]	Permanent
Cellular immune deficiencies	Permanent	Read v2 codes<br>ICD-10 codes[1]	Permanent
	Aplastic anaemia	Read v2 codes<br>ICD-10 codes[1]	24 months
	Other/unspecified cellular immune deficiences	Read v2 codes<br>ICD-10 codes[1]	90 days
Bone marrow transplants	Allogenic or autologous stem cell transplant	Read v2 codes<br>ICD-10 codes[1]<br>OPCS procedure codes[1]	24 months
Immunosuppressive therapies for solid organ transplant	Solid organ transplant	Read v2 codes<br>ICD-10 codes[1]<br>OPCS procedure codes[1]	Permanent
Chemotherapy or radiotherapy		Read v2 codes<br>Prescriptions<br>ICD-10 codes[1]<br>OPCS procedure codes[1]	1 year unless a record of end of therapy, in which case 6 months
Oral corticosteroids	Short term high-dose corticosteroids >40mg prednisolone per day for more than 1 week	Prescriptions	3 months
	Long term lower dose corticosteroids (>20mg prednisolone per day for more than 14 days	Prescriptions	3 months
Biological therapies	Abatacept, adalimumab, aflibercept, alemtuzumab, anakinra, apremilast, thymoglobulin, baricitinib, basiliximab, belatacept, bevacizumab, bortezomib, brentuximab, canakinumab, cetuximab, certolizumab, daclizumab, dasatinib, eculizumab, etanercept, everolimus, fingolimod, golimumab, idelalisib, imatinib, infliximab, ipilimumab, natalizumam, nilotinib, nivolumab, obinutuzumab, ofatumumab, panitumumab, pembrolizumab, pertuzumab, rituximab, secukinumab, temsirolimus, tocilizumab, tofacitinib, trastuzumab, ustekinumab, vedolizumab.	Prescriptions	3 months before first ever prescription[2] and 12 months following each prescription

For peer review only

Other immunosuppressant medications	Methotrexate >25mg per week; Azathioprine >3.0mg/kg/day; 6-mercaptopurine >1.5mg/kg/day; Corticosteroid injections; other DMARDS; other immunosuppressant medications	Prescriptions	3 months before first ever prescription[2] and 3 months following each prescription

1. For patients with available linkage to Hospital Episode Statistics (HES)
2. To reflect standard practice of initiation in secondary care.

For peer review only

STROBE Statement—Checklist of items that should be included in reports of ***cohort studies***

	Item No	Recommendation	Page
**Title and abstract**	1	(*a*) Indicate the study's design with a commonly used term in the title or the abstract	1
		(*b*) Provide in the abstract an informative and balanced summary of what was done and what was found	2
**Introduction**			
Background/rationale	2	Explain the scientific background and rationale for the investigation being reported	4
Objectives	3	State specific objectives, including any prespecified hypotheses	4
**Methods**			
Study design	4	Present key elements of study design early in the paper	6
Setting	5	Describe the setting, locations, and relevant dates, including periods of recruitment, exposure, follow-up, and data collection	5-6
Participants	6	(*a*) Give the eligibility criteria, and the sources and methods of selection of participants. Describe methods of follow-up	5
		(*b*) For matched studies, give matching criteria and number of exposed and unexposed	N/A
Variables	7	Clearly define all outcomes, exposures, predictors, potential confounders, and effect modifiers. Give diagnostic criteria, if applicable	5-6
Data sources/ measurement	8*	For each variable of interest, give sources of data and details of methods of assessment (measurement). Describe comparability of assessment methods if there is more than one group	*5-6*
Bias	9	Describe any efforts to address potential sources of bias	6
Study size	10	Explain how the study size was arrived at	5
Quantitative variables	11	Explain how quantitative variables were handled in the analyses. If applicable, describe which groupings were chosen and why	5-6
Statistical methods	12	(*a*) Describe all statistical methods, including those used to control for confounding	6
		(*b*) Describe any methods used to examine subgroups and interactions	N/A
		(*c*) Explain how missing data were addressed	5
		(*d*) If applicable, explain how loss to follow-up was addressed	N/A
		(*e*) Describe any sensitivity analyses	6
**Results**			
Participants	13*	(a) Report numbers of individuals at each stage of study—eg numbers potentially eligible, examined for eligibility, confirmed eligible, included in the study, completing follow-up, and analysed	8
		(b) Give reasons for non-participation at each stage	8
		(c) Consider use of a flow diagram	N/A

For peer review only

Descriptive data	14*	(a) Give characteristics of study participants (eg demographic, clinical, social) and information on exposures and potential confounders	8-9
		(b) Indicate number of participants with missing data for each variable of interest	N/A
		(c) Summarise follow-up time (eg, average and total amount)	8
Outcome data	15*	Report numbers of outcome events or summary measures over time	8-9
Main results	16	(*a*) Give unadjusted estimates and, if applicable, confounder-adjusted estimates and their precision (eg, 95% confidence interval). Make clear which confounders were adjusted for and why they were included	8-9
		(*b*) Report category boundaries when continuous variables were categorized	N/A
		(*c*) If relevant, consider translating estimates of relative risk into absolute risk for a meaningful time period	8-9
Other analyses	17	Report other analyses done—eg analyses of subgroups and interactions, and sensitivity analyses	8-9
**Discussion**			
Key results	18	Summarise key results with reference to study objectives	10
Limitations	19	Discuss limitations of the study, taking into account sources of potential bias or imprecision. Discuss both direction and magnitude of any potential bias	10-11
Interpretation	20	Give a cautious overall interpretation of results considering objectives, limitations, multiplicity of analyses, results from similar studies, and other relevant evidence	11
Generalisability	21	Discuss the generalisability (external validity) of the study results	11
**Other information**			
Funding	22	Give the source of funding and the role of the funders for the present study and, if applicable, for the original study on which the present article is based	12

*Give information separately for exposed and unexposed groups.

Note: An Explanation and Elaboration article discusses each checklist item and gives methodological background and published examples of transparent reporting. The STROBE checklist is best used in conjunction with this article (freely available on the Web sites of PLoS Medicine at http://www.plosmedicine.org/, Annals of Internal Medicine at http://www.annals.org/, and Epidemiology at http://www.epidem.com/). Information on the STROBE Initiative is available at http://www.strobe-statement.org.

For peer review only

BMJ Open

Safety of inadvertent administration of live zoster vaccine to immunosuppressed individuals in a UK-based observational cohort analysis

Journal:	*BMJ Open*
Manuscript ID	bmjopen-2019-034886.R2
Article Type:	Original research
Date Submitted by the Author:	03-Jan-2020
Complete List of Authors:	Grint, Daniel; London School of Hygiene and Tropical Medicine, Infectious Disease Epidemiology<br>McDonald, Helen; London School of Hygiene and Tropical Medicine, Infectious Disease Epidemiology<br>Walker, Jemma; London School of Hygiene and Tropical Medicine, Faculty of Epidemiology and Population Health; Public Health England, Statistics, Modelling and Economics Department<br>Amirthalingam, Gayatri; Public Health England, Immunisation and Countermeasures Division<br>Andrews, Nick; Public Health England, Statistics, Modelling and Economics Department; London School of Hygiene and Tropical Medicine, Infectious Disease Epidemiology<br>Thomas, Sara; London School of Hygiene and Tropical Medicine, Infectious Disease Epidemiology
**Primary Subject Heading**:	Public health
Secondary Subject Heading:	Epidemiology
Keywords:	Geriatric dermatology < DERMATOLOGY, IMMUNOLOGY, Public health < INFECTIOUS DISEASES, PUBLIC HEALTH, Adverse events < THERAPEUTICS, Infectious diseases & infestations < DERMATOLOGY

SCHOLARONE™ Manuscripts

I, the Submitting Author has the right to grant and does grant on behalf of all authors of the Work (as defined in the below author licence), an exclusive licence and/or a non-exclusive licence for contributions from authors who are: i) UK Crown employees; ii) where BMJ has agreed a CC-BY licence shall apply, and/or iii) in accordance with the terms applicable for US Federal Government officers or employees acting as part of their official duties; on a worldwide, perpetual, irrevocable, royalty-free basis to BMJ Publishing Group Ltd ("BMJ") its licensees and where the relevant Journal is co-owned by BMJ to the co-owners of the Journal, to publish the Work in this journal and any other BMJ products and to exploit all rights, as set out in our licence.

The Submitting Author accepts and understands that any supply made under these terms is made by BMJ to the Submitting Author unless you are acting as an employee on behalf of your employer or a postgraduate student of an affiliated institution which is paying any applicable article publishing charge ("APC") for Open Access articles. Where the Submitting Author wishes to make the Work available on an Open Access basis (and intends to pay the relevant APC), the terms of reuse of such Open Access shall be governed by a Creative Commons licence – details of these licences and which Creative Commons licence will apply to this Work are set out in our licence referred to above.

Other than as permitted in any relevant BMJ Author's Self Archiving Policies, I confirm this Work has not been accepted for publication elsewhere, is not being considered for publication elsewhere and does not duplicate material already published. I confirm all authors consent to publication of this Work and authorise the granting of this licence.

For peer review only

Safety of inadvertent administration of live zoster vaccine to immunosuppressed individuals in a UK-based observational cohort analysis

For peer review only

Authors: Daniel J Grint (0000-0001-8001-6544),[1] Helen I McDonald (0000-0003-0576-2015),[1] Jemma L Walker,[1,2] Gayatri Amirthalingam,[3] Nick Andrews,[1,2] Sara L Thomas.[1]

1. Department of Infectious Disease Epidemiology, London School of Hygiene & Tropical Medicine, London, UK

Daniel J Grint assistant professor, Helen I McDonald clinical research fellow, Jemma L Walker research fellow, Nick Andrews honorary professor, Sara L Thomas professor (retired).

2. Statistics, Modelling and Economics Department, Public Health England, London, UK

Jemma L Walker statistician, Nick Andrews senior statistician.

3. Immunisation and Countermeasures Division, National Infection Service, Public Health England, London, UK

Gayatri Amirthalingam consultant epidemiologist.

Correspondence to: Helen McDonald, London School of Hygiene & Tropical Medicine, Keppel Street, London, WC1E 7HT. Helen.mcdonald@lshtm.ac.uk Tel: 020 7612 7921 Fax: N/A

Article type: Research

For peer review only - http://bmjopen.bmj.com/site/about/guidelines.xhtml

Abstract

Objectives:

To investigate the safety of live attenuated varicella zoster vaccination when administered to immunosuppressed individuals.

Design:

Prospective observational cohort study.

Setting:

The study used anonymised data from the Clinical Practice Research Datalink (CPRD), comprising a representative sample of routinely collected primary care data in England between 2013 and 2017, and linked Hospital Episode Statistics (HES) data.

Participants:

168,767 individuals age-eligible for varicella zoster vaccination registered at a general practice in England contributing data to CPRD.

Main outcome measures:

Electronic health records indicating immunosuppression, zoster vaccination, diagnoses of specific varicella-zoster virus (VZV)-related disease and non-specific rash/encephalitis compatible with VZV-related disease.

Results:

Between 1st September 2013 and 31st August 2017, a period of immunosuppression was identified for 9,093/168,767 (5·4%; 95% CI: 5·3-5·5%) individuals age-eligible for zoster vaccination. The overall rate of vaccination while immunosuppressed was 1,742/5,251 (33·2 per 100 adjusted person years at risk; 95% CI: 31·9-34·5). Follow-up of the 1,742 individuals who were inadvertently vaccinated while immunosuppressed identified only two cases of VZV-related disease within 8 weeks of vaccination (0·1%; 95% CI: 0·01-0·4%), both primary care diagnoses of “shingles”, neither with a related hospital admission.

Conclusions:

Despite evidence of inadvertent vaccination of immunosuppressed individuals with live zoster vaccination, there is a lack of evidence of severe consequences including hospitalisation. This should

For peer review only - http://bmjopen.bmj.com/site/about/guidelines.xhtml

reassure primary care staff and encourage vaccination of mildly immunosuppressed individuals who do not meet current thresholds for contraindication. These findings support a review of the extent to which live zoster vaccination is contraindicated among the immunosuppressed.

For peer review only

For peer review only - http://bmjopen.bmj.com/site/about/guidelines.xhtml

Strengths and limitations

- This study investigated the safety of live zoster vaccination during immunosuppression in a large national cohort using electronic health records.
- It is the first study to cover the full profile of causes of immunosuppression listed as contraindications to vaccination in UK national guidance, ascertained from multiple primary and secondary care sources.
- Both primary and secondary care records were used for thorough ascertainment of VZV-related disease, including a sensitivity analysis ascertaining non-specific rash or encephalitis of unspecified aetiology.
- Vaccination rates were analysed using only year of birth for age-eligibility, and so the denominator was adjusted for birth cohorts with partial eligibility.
- Immunosuppression was not distinguished according to severity, but clinicians may have vaccinated selectively and caution would be needed in applying these findings outside of current vaccination practice.

For peer review only

Introduction

Herpes zoster (shingles) is a common and painful disease caused by reactivation of varicella zoster virus (VZV), with debilitating complications including post-herpetic neuralgia. Live-attenuated zoster vaccine was introduced for immunocompetent adults aged 70-79 years in England in 2013, delivered in primary care.(1) The herpes zoster vaccination programme in England was found to have a population impact equivalent to approximately 17,000 fewer episodes of herpes zoster and 3,300 fewer episodes of postherpetic neuralgia among 5·5 million eligible individuals in the first 3 years of the programme.(2)

Immunosuppression is associated with a high burden of zoster and its complications,(3, 4) and there have been calls to consider vaccination for this population.(5) However, live zoster vaccine is currently contraindicated in immunosuppression as it may cause VZV-related disease.(1) High-profile case reports of fatal vaccine-related disease among severely immunosuppressed individuals have caused concern and may have contributed to declining vaccine coverage.(6, 7) Understanding the safety of live vaccination during immunosuppression is important to support guidance on use of the vaccine, to ensure that individuals who can safely benefit from the vaccine are enabled to do so.

A new vaccine which is recombinant rather than containing live virus could offer a safer alternative for immunosuppressed individuals without the risk of vaccine-related disease, and has been found to be effective among patients with autologous hematopoietic stem cell transplantation (HSCT).(8) However, supplies are currently unable to meet global demands. The Joint Committee on Vaccination and Immunisation has recommended use of the recombinant vaccine for individuals with immunosuppression. Understanding the safety of live vaccination for typical causes of immunosuppression will be important to prioritise use of limited supplies of the new recombinant vaccine. When recombinant vaccine is used in cases for which live vaccine is contraindicated, this may introduce potential for confusion and it will be even more important to understand the consequences of inadvertent live vaccination during immunosuppression.

For peer review only - http://bmjopen.bmj.com/site/about/guidelines.xhtml

This study aimed to investigate the frequency and consequences of live zoster vaccination during immunosuppression among a large UK cohort from 2013 to 2017.

For peer review only

For peer review only - http://bmjopen.bmj.com/site/about/guidelines.xhtml

Methods

Data source

This study used anonymised data from the Clinical Practice Research Datalink (CPRD). The data include information on year of birth, medical diagnoses (version 2 Read codes), prescriptions and vaccinations. For 60% of individuals, records are pre-linked to anonymised hospitalisation data (Hospital Episode Statistics, HES). HES-linked data for inpatient admissions (International Classification of Disease, ICD-10 codes) and procedures (OPCS-4 Classification of Procedures codes) were used to supplement identification of immunosuppressed individuals and VZV-related disease.

Study population

Immunosuppressed individuals age-eligible for zoster vaccination, active in CPRD from September 2013 to August 2017 and registered with a CPRD practice for at least a year before study entry, were included.

Age eligibility for zoster vaccination has differed each year since the vaccination introduction. As month of birth was not available, individuals born in years for which ≥67% of the population would have been eligible for vaccination were included (Appendix A1). Birth cohorts were defined as ‘maiden cohorts’ in the first year for which they were age-eligible for vaccination.

Periods of immunosuppression were identified using Read codes and prescription records from CPRD, plus ICD-10 codes and OPCS codes in linked HES data using algorithms previously described.(9) Immunosuppression was defined based on contraindications to live zoster vaccination described in national guidance.(1) The time periods assigned to each immunosuppressing condition or medication type, and dose thresholds for relevant medications, are described in Appendix A2. For prescription records missing dose, the median was imputed according to category of age and sex, in line with previous zoster studies.(4)

Vaccination status and varicella zoster virus-related disease

For peer review only - http://bmjopen.bmj.com/site/about/guidelines.xhtml

Individuals were followed to the first positive record of zoster vaccination. If this indicated that the vaccine was delivered by another health care provider the individual was excluded from the cohort, as timing of vaccination could not be determined (N=29).

Evidence of VZV-related disease in primary or secondary care records was assessed during the 8 weeks following a vaccination given while immunosuppressed. For the primary analysis, only specific diagnoses of VZV disease were included. Sensitivity analysis also included any rash that was unspecified or compatible with VZV, and acute encephalitis of unspecified aetiology. For individuals with HES-linkage, any diagnosis recorded within 8 weeks following vaccination was used to supplement identification of VZV-related disease. Codelists are available at https://datacompass.lshtm.ac.uk/1336/.

Statistical methods

An open prospective cohort study design was used whereby individuals exited and re-entered the cohort according to time-varying immunosuppression status. Follow-up started at 1st September of the study year in which the individual was age-eligible for vaccination and ended at the earliest of death, date of transfer out of practice, practice last collection date, elapsed age-eligibility for zoster vaccination, resolution of immunosuppression, or 31st August 2017.

The vaccination rate was calculated by total person years at risk (PYAR) while immunosuppressed with adjustment to account for age-eligibility uncertainty from unknown month of birth (Appendix A1). Cumulative uptake of zoster vaccine was computed stratified by treatment cohort and programme year.

The number of vaccinated immunosuppressed individuals who developed VZV-related disease in the subsequent eight weeks was described. In sensitivity analysis, disease in the first week following vaccination was excluded.

Statistical analysis was performed using STATA version 14·2 and SAS version 9.4.

For peer review only - http://bmjopen.bmj.com/site/about/guidelines.xhtml

Ethics approval

The study was approved by the Independent Scientific Advisory Group of the CPRD (ISAC reference 18_218A) and the London School of Hygiene and Tropical Medicine Ethics Committee (reference 16298). The amended ISAC protocol was made available to reviewers.

Patient and public involvement

Patients and the public were not involved in the design or planning of the study.

For peer review only

For peer review only - http://bmjopen.bmj.com/site/about/guidelines.xhtml

Results

Between 1st September 2013 and 31st August 2017 data were available for 168,767 individuals age-eligible for vaccination, of whom 89,416 (53·0%) were female and 76,337 (45·2%) in the catch-up cohort. A period of immunosuppression while age-eligible for vaccination was identified for 9,093/168,767 (5·4%; 95% CI: 5·3-5·5%).

1,742 individuals were vaccinated during a period of immunosuppression. Adjusting person years at risk while immunosuppressed to account for age-eligibility uncertainty, the overall rate of vaccination during immunosuppression was 1,742/5,251 (33·2 per 100 adjusted person years at risk; 95% CI: 31·9-34·5). Figure 1 shows the cumulative uptake of zoster vaccine by programme year overall, in maiden years of eligibility and by cohort. Cumulative uptake was higher in programme years 3 and 4 when restricted to maiden years of eligibility. Cumulative uptake was highest in programme years 1 and 2 for both the routine and catch-up cohorts.

Among those vaccinated while immunosuppressed the most common underlying cause was chemotherapy (55·3%), followed by other immunosuppressant therapies including biologics (13·2%), multiple indications (11·4%), and steroid drug use (11·3%). 47 (2·7%) had a permanent cause of immunosuppression. 368 (21·1%) were immunosuppressed for the duration of follow-up; median follow-up 32·2 months (IQR: 19·7-48·0). Vaccination took place during the final four weeks of a defined period of immunosuppression for 138/1,742 (7·9%).

In the eight weeks following vaccination 2/1,742 (0·1%; 95% CI: 0·01-0·4%) had a diagnosis of "shingles" recorded in primary care. Both individuals had HES-linkage available, however, neither had a related hospital admission. One of these cases occurred within 7 days of vaccination.

Using a broader definition including non-specific rash or encephalitis identified a further 23 possible cases of VZV-related disease (in total 25/1,742 (1·4%; 95% CI: 0·9-2·1%)). All of these were instances

For peer review only - http://bmjopen.bmj.com/site/about/guidelines.xhtml

of unspecified rash in primary care, and there were no cases of encephalitis. In total, 22/25 (88%) had HES-linkage available, however, none had a related hospital admission recorded. Five of the broader definition cases occurred within 7 days of vaccine administration.

Chemotherapy was the cause of immunosuppression for the majority of cases who developed specific or non-specific potentially vaccine-related disease (15/25 (60%)). The remaining cases included immunosuppression by oral steroid use, other immunosuppressant medications, leukaemia, and organ transplant.

For peer review only

For peer review only - http://bmjopen.bmj.com/site/about/guidelines.xhtml

Discussion

This is the first study to investigate the safety of live zoster vaccination across the range of contraindicating immunosuppressive conditions. Our study identified 1,742 individuals vaccinated while immunosuppressed and two subsequent cases with a diagnosis of "shingles", with no related hospitalisations, and no cases of encephalitis.

A key strength of this study is the thorough ascertainment of both immunosuppression and VZV-related disease using linked primary and secondary care data for a large, representative cohort with a range of immunosuppressive conditions.

The study has limitations. A key limitation is that month of birth was not available for precise identification of age-eligibility. If immunosuppressed individuals in a birth cohort with 67% eligibility were vaccinated while not age-eligible, rates of vaccination in immunosuppression would be overestimated. There also remains uncertainty in defining time-periods of immunosuppression and in imputing missing dose data for medications, which may result in under- or over-ascertainment of immunosuppression.

It is possible that VZV-related disease may have been under-ascertained, either because patients did not attend healthcare or due to non-specific coding. A US study reported that 95% of patients aged over 60 years had attended healthcare when they experienced zoster disease,(10) and this might be expected to be higher among immunosuppressed individuals in a setting with universal healthcare. This study used both primary care and secondary care records to ascertain VZV-related disease, an approach which has previously been found to generate plausible estimates of zoster incidence among the older general population.(11) The sensitivity analysis was also designed to ascertain possible cases of VZV-related disease which may have been coded non-specifically as rash or encephalitis. Conversely, as this population has a high baseline risk of naturally occurring shingles, we may have

For peer review only - http://bmjopen.bmj.com/site/about/guidelines.xhtml

over-estimated the risk attributable to vaccination, particularly when including cases within 7 days of vaccination.

Finally, while our definitions of immunosuppression followed national guidance, we could not replace clinical judgement on severity or timing of immunosuppression, and the study was not powered to assess safety according to type of immunosuppression.(1) Clinicians may have selectively, rather than inadvertently, vaccinated individuals at lower risk of vaccine-related disease, resulting in a 'healthy vaccinee' effect, and caution would be needed in generalising these findings outside of current practice in the context of guidance on contraindications. However, the most frequent contraindication was chemotherapy, and vaccinations did not occur disproportionately towards the end of a period of immunosuppression, suggesting that vaccinations were not all at the margins of the guidance.

Our safety findings are consistent with previous studies of live zoster vaccination among patients with selected immunosuppressive conditions.(12-14) Studies showing that VZV-specific immunity may persist or even be boosted by vaccination during cell-mediated immunosuppression,(15-17) further support the plausibility of residual immunity against vaccine-related disease despite immunosuppression.

Rates of zoster vaccination during immunosuppression were high, and similar in routine and catch-up cohorts. To our knowledge, this is the first study to calculate rates of live zoster vaccination across this range of immunosuppressing conditions. A zoster vaccine effectiveness study of Medicare beneficiaries in the US included 140,925 individuals with a diagnosis of leukaemia, lymphoma or HIV, of whom 4,469 (3.2%) were vaccinated while immunosuppressed, comparable to the overall study vaccine uptake (29,785/766,330; 3.9%), suggesting that live zoster vaccination despite immunosuppression is not unique to the UK setting.(16) In our study, analysis restricted to maiden years of eligibility suggests that the apparent decline in vaccination rates after year 2 is partly a cohort effect, whereby people who were unvaccinated despite previous eligibility were less likely to be vaccinated subsequently. This could be due to an initial decision not to vaccinate continuing over

For peer review only - http://bmjopen.bmj.com/site/about/guidelines.xhtml

subsequent years of eligibility, or a greater focus on vaccination for newly eligible patients. Increasingly detailed guidance over time may also have helped reduce inadvertent vaccination.

Among this large cohort with a range of immunosuppressing conditions we found no evidence of severe VZV-related disease following live zoster vaccination while immunosuppressed. This should reassure clinicians, and encourage vaccination of mildly immunosuppressed individuals who do not meet current thresholds for contraindication, especially in the current context of declining uptake of the national programme.(18) These findings support a review of the extent to which live zoster vaccination is contraindicated among the immunosuppressed population. Further research is needed to identify any patient groups for whom recombinant zoster vaccine should be prioritised once stocks become available in the UK.

For peer review only

Role of the funding source

The research was funded by the National Institute for Health Research (NIHR) Health Protection Research Unit (HPRU) in Immunisation at the London School of Hygiene and Tropical Medicine in partnership with Public Health England (PHE). The funders had no role in the study design, collection analysis or interpretation of data, writing of the report nor the decision to submit the article for publication. The views expressed are those of the authors and not necessarily those of the NHS, the NIHR, the Department of Health and Social Care, or PHE.

Contributor and guarantor information:

SLT had the idea for the study. SLT, JLW, GA and NA obtained the data. All authors contributed to the design of the study. DJG analysed the data. All authors contributed to the interpretation of results. DJG and HIM drafted the manuscript and all authors revised it critically. All authors approved the final version to be published and agree to be accountable for all aspects of the work. DJG is the guarantor. The corresponding author attests that all listed authors meet authorship criteria and that no others meeting the criteria have been omitted.

Transparency declaration: DJG affirms that the manuscript is an honest, accurate, and transparent account of the study being reported; that no important aspects of the study have been omitted; and that any discrepancies from the study as originally planned (and, if relevant, registered) have been explained.

Competing interests declaration: All authors have completed the ICMJE uniform disclosure form at www.icmje.org/coi_disclosure.pdf and declare: DJG, JLW, NA, and SLT had financial support from the National Institute for Health Research (NIHR) Health Protection Research Unit (HPRU) in Immunisation for the submitted work; the Public Health England Immunisation Department has provided vaccine manufacturers with post-marketing surveillance reports on pneumococcal and meningococcal infection which the companies are required to submit to the UK Licensing Authority in compliance with their Risk Management Strategy, and a cost recovery charge is made for these

For peer review only

For peer review only - http://bmjopen.bmj.com/site/about/guidelines.xhtml

reports; no other financial relationships with any organisations that might have an interest in the submitted work in the previous three years; no other relationships or activities that could appear to have influenced the submitted work.

Licence for publication

The Corresponding Author has the right to grant on behalf of all authors and does grant on behalf of all authors, a worldwide licence to the Publishers and its licensees in perpetuity, in all forms, formats and media (whether known now or created in the future), to i) publish, reproduce, distribute, display and store the Contribution, ii) translate the Contribution into other languages, create adaptations, reprints, include within collections and create summaries, extracts and/or, abstracts of the Contribution, iii) create any other derivative work(s) based on the Contribution, iv) to exploit all subsidiary rights in the Contribution, v) the inclusion of electronic links from the Contribution to third party material where-ever it may be located; and, vi) licence any third party to do any or all of the above.

Data sharing statement

These data were obtained from the Clinical Practice Research Datalink, provided by the UK Medicines and Healthcare products Regulatory Agency. The authors' licence for using these data does not allow sharing of raw data with third parties. Information about access to Clinical Practice Research Datalink data is available here: https://www.cprd.com/research-applications

Codelists for this study are available at https://datacompass.lshtm.ac.uk/1336/.

For peer review only

References

1. Public Health England. Immunisation against Infectious Disease (the Green Book). Chapter 28a: Shingles (herpes zoster) 2016. Available from: https://assets.publishing.service.gov.uk/government/uploads/system/uploads/attachment_data/file/503773/2905109_Green_Book_Chapter_28a_v3_0W.PDF
2. Amirthalingam G, Andrews N, Keel P, Mullett D, Correa A, de Lusignan S, et al. Evaluation of the effect of the herpes zoster vaccination programme 3 years after its introduction in England: a population-based study. The lancet Public Health. 2018;3(2):e82-e90.
3. Yanni EA, Ferreira G, Guennec M, El Hahi Y, El Ghachi A, Haguinet F, et al. Burden of herpes zoster in 16 selected immunocompromised populations in England: a cohort study in the Clinical Practice Research Datalink 2000-2012. BMJ open. 2018;8(6):e020528.
4. Forbes HJ, Bhaskaran K, Thomas SL, Smeeth L, Clayton T, Mansfield K, et al. Quantification of risk factors for postherpetic neuralgia in herpes zoster patients: A cohort study. Neurology. 2016;87(1):94-102.
5. Oxman MN, Schmader KE. Editorial commentary: zoster vaccine in immunocompromised patients: time to reconsider current recommendations. Clin Infect Dis. 2014;59(7):920-2.
6. Costa E, Buxton J, Brown J, Templeton KE, Breuer J, Johannessen I. Fatal disseminated varicella zoster infection following zoster vaccination in an immunocompromised patient. BMJ Case Rep. 2016;2016.
7. Alexander KE, Tong PL, Macartney K, Beresford R, Sheppeard V, Gupta M. Live zoster vaccination in an immunocompromised patient leading to death secondary to disseminated varicella zoster virus infection. Vaccine. 2018;36(27):3890-3.
8. Bastidas A, de la Serna J, El Idrissi M, Oostvogels L, Quittet P, Lopez-Jimenez J, et al. Effect of Recombinant Zoster Vaccine on Incidence of Herpes Zoster After Autologous Stem Cell Transplantation: A Randomized Clinical Trial. JAMA. 2019;322(2):123-33.
9. Walker JL, Andrews NJ, Amirthalingam G, Forbes H, Langan SM, Thomas SL. Effectiveness of herpes zoster vaccination in an older United Kingdom population. Vaccine. 2018;36(17):2371-7.
10. Lu PJ, Euler GL, Jumaan AO, Harpaz R. Herpes zoster vaccination among adults aged 60 years or older in the United States, 2007: uptake of the first new vaccine to target seniors. Vaccine. 2009;27(6):882-7.
11. Jain A, van Hoek AJ, Walker JL, Forbes HJ, Langan SM, Root A, et al. Inequalities in zoster disease burden: a population-based cohort study to identify social determinants using linked data from the U.K. Clinical Practice Research Datalink. The British journal of dermatology. 2018;178(6):1324-30.
12. Zhang J, Xie F, Delzell E, Chen L, Winthrop KL, Lewis JD, et al. Association between vaccination for herpes zoster and risk of herpes zoster infection among older patients with selected immune-mediated diseases. Jama. 2012;308(1):43-9.
13. Cheetham TC, Marcy SM, Tseng HF, Sy LS, Liu IL, Bixler F, et al. Risk of Herpes Zoster and Disseminated Varicella Zoster in Patients Taking Immunosuppressant Drugs at the Time of Zoster Vaccination. Mayo Clin Proc. 2015;90(7):865-73.
14. Naidus E, Damon L, Schwartz BS, Breed C, Liu C. Experience with use of Zostavax((R)) in patients with hematologic malignancy and hematopoietic cell transplant recipients. Am J Hematol. 2012;87(1):123-5.
15. Tseng HF, Tartof S, Harpaz R, Luo Y, Sy LS, Hetcher RC, et al. Vaccination against zoster remains effective in older adults who later undergo chemotherapy. Clin Infect Dis. 2014;59(7):913-9.
16. Langan SM, Smeeth L, Margolis DJ, Thomas SL. Herpes zoster vaccine effectiveness against incident herpes zoster and post-herpetic neuralgia in an older US population: a cohort study. PLoS medicine. 2013;10(4):e1001420.

For peer review only - http://bmjopen.bmj.com/site/about/guidelines.xhtml

17. Benson CA, Andersen JW, Macatangay BJ, Mailliard RB, Rinaldo Jr CR, Read S, et al. Safety and Immunogenicity of Zoster Vaccine Live in Human Immunodeficiency Virus–Infected Adults With CD4+ Cell Counts> 200 Cells/mL Virologically Suppressed on Antiretroviral Therapy. Clinical Infectious Diseases. 2018;67(11):1712-9.
18. Public Health England. Herpes zoster (shingles) immunisation programme 2017 to 2018: evaluation report Health Protection Report: PHE Publications gateway number:2018660; 2018.

For peer review only

For peer review only - http://bmjopen.bmj.com/site/about/guidelines.xhtml

Figure legend

Cumulative uptake of zoster vaccination while immunosuppressed, by vaccination programme year.

A) Stratified by maiden years of eligibility or overall eligibility. B) Stratified by routine or catch-up cohort.

For peer review only

For peer review only

For peer review only - http://bmjopen.bmj.com/site/about/guidelines.xhtml

Panel A: Overall uptake, and limited to maiden year eligibility

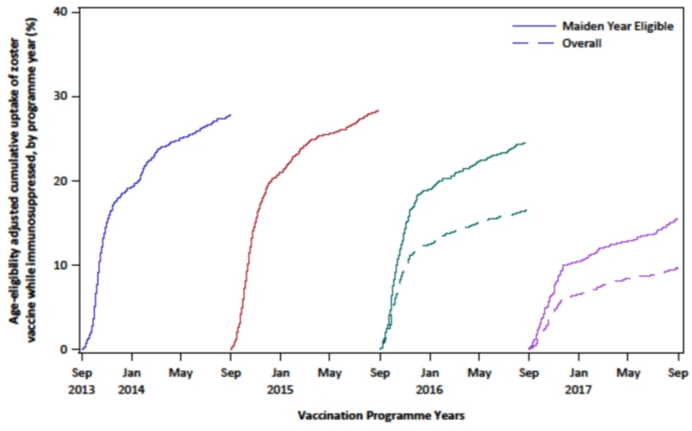

Panel B: Routine and catch-up birth cohorts

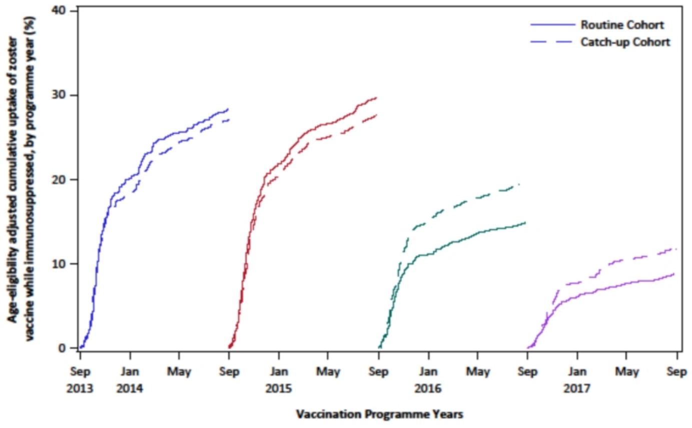

209x296mm (300 x 300 DPI)

Appendices

Appendix A1: Approach to defining age-eligible cohorts for zoster vaccination

CPRD data are anonymised and includes year of birth but not day or month. This is a challenge for identifying the age-cohorts eligible for vaccination. Age eligibility for zoster vaccination has differed for each year of the vaccination programme since its introduction, as follows:

- *2013/14*: those aged 70 or 79 years on 1st Sept 2013;
- *2014/15:* those aged 70, 78 or 79 years on 1st Sept 2014;
- *2015/16*: those aged 70, 71, 72, 78 or 79 years on 1st Sept 2015;
- *2016/17*: those aged 70, 71, 72, 73, 78 or 79 years on 1st Sept 2016.

The distribution of zoster vaccine eligibility according to year of birth is detailed in Table A1, below. We included the following age cohorts over the four years of the study to ensure that at least 67% of the cohort was age-eligible in each year:

- Study year 1: 2013/14: years of birth 1943, 1934
- Study year 2: 2014/15: years of birth 1944, 1935, 1936
- Study year 3: 2015/16: years of birth 1943*, 1944*, 1945, 1936*, 1937
- Study year 4: 2016/17: years of birth 1943*, 1944*, 1945*, 1946, 1937*, 1938

  * Included if not previously vaccinated

Age-eligibility person years at risk adjustment

The rate of vaccination while immunosuppressed was calculated by adjusting the denominator person years at risk to account for age-eligibility uncertainty relating to unknown month of birth. For example, people born in 1943 were eligible if born prior to 2nd September (Table A1), so eligibility for those born in 1943 was assumed to be 67%. Therefore, the total person years at risk of people born in 1943 was adjusted by multiplying by 0.67. This process was followed for all years with partial age-eligibility.

For cumulative uptake analysis, those born in years with partial (67%) age-eligibility contributed 0.67 to the denominator.

For peer review only

Table A1: Summary of zoster vaccine eligibility by calendar year and year of birth

			Year 1: 09/2013-08/2014		Year 2: 09/2014-08/2015		Year 3: 09/2015-08/2016		Year 4: 09/2016-08/2017	
Year of birth	True day/month of birth	cohort	Age at 01/09	Eligible?	Age at 01/09	Eligible?	Age at 01/09	Eligible?	Age at 01/09*	Eligible?
1943	1st Jan-1st Sep	Routine	70	Eligible	71	Ineligible	72	Eligible	73	Eligible
1943	2nd Sep-31st Dec	Routine	69	Ineligible	70	Eligible	71	Eligible	72	Eligible
1944	1st Jan-1st Sep	Routine	69	Ineligible	70	Eligible	71	Eligible	72	Eligible
1944	2nd Sep-31st Dec	Routine	68	Ineligible	69	Ineligible	70	Eligible	71	Eligible
1945	1st Jan-1st Sep	Routine	68	Ineligible	69	Ineligible	70	Eligible	71	Eligible
1945	2nd Sep-31st Dec	Routine	67	Ineligible	68	Ineligible	69	Ineligible	70	Eligible
1946	1st Jan-1st Sep	Routine	67	Ineligible	68	Ineligible	69	Ineligible	70	Eligible
1946	2nd Sep-31st Dec	Routine	66	Ineligible	67	Ineligible	68	Ineligible	69	Ineligible*
1934	1st Jan-1st Sep	Catch up	79	Eligible	80	Ineligible	81	Ineligible	82	Ineligible
1934	2nd Sep-31st Dec	Catch up	78	Ineligible	79	Eligible	80	Ineligible	81	Ineligible
1935	1st Jan-1st Sep	Catch up	78	Ineligible	79	Eligible	80	Ineligible	81	Ineligible
1935	2nd Sep-31st Dec	Catch up	77	Ineligible	78	Eligible	79	Eligible	80	Ineligible
1936	1st Jan-1st Sep	Catch up	77	Ineligible	78	Eligible	79	Eligible	80	Ineligible
1936	2nd Sep-31st Dec	Catch up	76	Ineligible	77	Ineligible	78	Eligible	79	Eligible
1937	1st Jan-1st Sep	Catch up	76	Ineligible	77	Ineligible	78	Eligible	79	Eligible
1937	2nd Sep-31st Dec	Catch up	75	Ineligible	76	Ineligible	77	Ineligible	78	Eligible
1938	1st Jan-1st Sep	Catch up	75	Ineligible	76	Ineligible	77	Ineligible	78	Eligible
1938	2nd Sep-31st Dec	Catch up	74	Ineligible	75	Ineligible	76	Ineligible	77	Ineligible*

* From April 2017, rules changed so patients became eligible on the day they turned 70yrs (routine) or 78 years (catch up); those with existing eligibility who missed vaccination could still be offered the vaccine.

For peer review only

Appendix A2: Summary of definitions of periods of immunosuppression

**Immunosuppression category**	**Category includes**	**Code types used to identify immunosuppression**	**Time period defined as immunosuppressed following each record**
Haematological malignancies	Leukaemias, lymphomas, other lymphoproliferative disorders	Read v2 codes<br>ICD-10 codes[1]	24 months
HIV/AIDS		Read v2 codes<br>ICD-10 codes[1]	Permanent
Cellular immune deficiencies	Permanent	Read v2 codes<br>ICD-10 codes[1]	Permanent
	Aplastic anaemia	Read v2 codes<br>ICD-10 codes[1]	24 months
	Other/unspecified cellular immune deficiences	Read v2 codes<br>ICD-10 codes[1]	90 days
Bone marrow transplants	Allogenic or autologous stem cell transplant	Read v2 codes<br>ICD-10 codes[1]<br>OPCS procedure codes[1]	24 months
Immunosuppressive therapies for solid organ transplant	Solid organ transplant	Read v2 codes<br>ICD-10 codes[1]<br>OPCS procedure codes[1]	Permanent
Chemotherapy or radiotherapy		Read v2 codes<br>Prescriptions<br>ICD-10 codes[1]<br>OPCS procedure codes[1]	1 year unless a record of end of therapy, in which case 6 months
Oral corticosteroids	Short term high-dose corticosteroids >40mg prednisolone per day for more than 1 week	Prescriptions	3 months
	Long term lower dose corticosteroids (>20mg prednisolone per day for more than 14 days	Prescriptions	3 months
Biological therapies	Abatacept, adalimumab, aflibercept, alemtuzumab, anakinra, apremilast, thymoglobulin, baricitinib, basiliximab, belatacept, bevacizumab, bortezomib, brentuximab, canakinumab, cetuximab, certolizumab, daclizumab, dasatinib, eculizumab, etanercept, everolimus, fingolimod, golimumab, idelalisib, imatinib, infliximab, ipilimumab, natalizumam, nilotinib, nivolumab, obinutuzumab, ofatumumab, panitumumab, pembrolizumab, pertuzumab, rituximab, secukinumab, temsirolimus, tocilizumab, tofacitinib, trastuzumab, ustekinumab, vedolizumab.	Prescriptions	3 months before first ever prescription[2] and 12 months following each prescription

For peer review only

Other immunosuppressant medications	Methotrexate >25mg per week; Azathioprine >3.0mg/kg/day; 6-mercaptopurine >1.5mg/kg/day; Corticosteroid injections; other DMARDS; other immunosuppressant medications	Prescriptions	3 months before first ever prescription[2] and 3 months following each prescription

1. For patients with available linkage to Hospital Episode Statistics (HES)
2. To reflect standard practice of initiation in secondary care.

For peer review only

STROBE Statement—Checklist of items that should be included in reports of ***cohort studies***

	Item No	Recommendation	Page
**Title and abstract**	1	(*a*) Indicate the study's design with a commonly used term in the title or the abstract	1
		(*b*) Provide in the abstract an informative and balanced summary of what was done and what was found	2
**Introduction**			
Background/rationale	2	Explain the scientific background and rationale for the investigation being reported	4
Objectives	3	State specific objectives, including any prespecified hypotheses	4
**Methods**			
Study design	4	Present key elements of study design early in the paper	6
Setting	5	Describe the setting, locations, and relevant dates, including periods of recruitment, exposure, follow-up, and data collection	5-6
Participants	6	(*a*) Give the eligibility criteria, and the sources and methods of selection of participants. Describe methods of follow-up	5
		(*b*) For matched studies, give matching criteria and number of exposed and unexposed	N/A
Variables	7	Clearly define all outcomes, exposures, predictors, potential confounders, and effect modifiers. Give diagnostic criteria, if applicable	5-6
Data sources/ measurement	8*	For each variable of interest, give sources of data and details of methods of assessment (measurement). Describe comparability of assessment methods if there is more than one group	*5-6*
Bias	9	Describe any efforts to address potential sources of bias	6
Study size	10	Explain how the study size was arrived at	5
Quantitative variables	11	Explain how quantitative variables were handled in the analyses. If applicable, describe which groupings were chosen and why	5-6
Statistical methods	12	(*a*) Describe all statistical methods, including those used to control for confounding	6
		(*b*) Describe any methods used to examine subgroups and interactions	N/A
		(*c*) Explain how missing data were addressed	5
		(*d*) If applicable, explain how loss to follow-up was addressed	N/A
		(*e*) Describe any sensitivity analyses	6
**Results**			
Participants	13*	(a) Report numbers of individuals at each stage of study—eg numbers potentially eligible, examined for eligibility, confirmed eligible, included in the study, completing follow-up, and analysed	8
		(b) Give reasons for non-participation at each stage	8
		(c) Consider use of a flow diagram	N/A

For peer review only - http://bmjopen.bmj.com/site/about/guidelines.xhtml

Descriptive data	14*	(a) Give characteristics of study participants (eg demographic, clinical, social) and information on exposures and potential confounders	8-9
		(b) Indicate number of participants with missing data for each variable of interest	N/A
		(c) Summarise follow-up time (eg, average and total amount)	8
Outcome data	15*	Report numbers of outcome events or summary measures over time	8-9
Main results	16	(*a*) Give unadjusted estimates and, if applicable, confounder-adjusted estimates and their precision (eg, 95% confidence interval). Make clear which confounders were adjusted for and why they were included	8-9
		(*b*) Report category boundaries when continuous variables were categorized	N/A
		(*c*) If relevant, consider translating estimates of relative risk into absolute risk for a meaningful time period	8-9
Other analyses	17	Report other analyses done—eg analyses of subgroups and interactions, and sensitivity analyses	8-9
**Discussion**			
Key results	18	Summarise key results with reference to study objectives	10
Limitations	19	Discuss limitations of the study, taking into account sources of potential bias or imprecision. Discuss both direction and magnitude of any potential bias	10-11
Interpretation	20	Give a cautious overall interpretation of results considering objectives, limitations, multiplicity of analyses, results from similar studies, and other relevant evidence	11
Generalisability	21	Discuss the generalisability (external validity) of the study results	11
**Other information**			
Funding	22	Give the source of funding and the role of the funders for the present study and, if applicable, for the original study on which the present article is based	12

*Give information separately for exposed and unexposed groups.

Note: An Explanation and Elaboration article discusses each checklist item and gives methodological background and published examples of transparent reporting. The STROBE checklist is best used in conjunction with this article (freely available on the Web sites of PLoS Medicine at http://www.plosmedicine.org/, Annals of Internal Medicine at http://www.annals.org/, and Epidemiology at http://www.epidem.com/). Information on the STROBE Initiative is available at http://www.strobe-statement.org.

For peer review only

For peer review only - http://bmjopen.bmj.com/site/about/guidelines.xhtml
